# Flexible use of copula‐type model for dose‐finding in drug combination clinical trials

**DOI:** 10.1111/biom.13510

**Published:** 2021-08-01

**Authors:** Koichi Hashizume, Jun Tshuchida, Takashi Sozu

**Affiliations:** ^1^ Department of Information and Computer Technology, Graduate School of Engineering Tokyo University of Science Tokyo Japan; ^2^ Global Biometrics and Data Science Bristol Myers Squibb K.K Tokyo Japan; ^3^ Department of Culture and Information Science, Faculty of Culture and Information Science Doshisha University Kyoto Japan; ^4^ Department of Information and Computer Technology, Faculty of Engineering Tokyo University of Science Tokyo Japan

**Keywords:** combining drugs, copula regression, maximum tolerated dose, oncology, phase I trial, synergistic toxicity

## Abstract

Identification of the maximum tolerated dose combination (MTDC) of cancer drugs is an important objective in phase I oncology trials. Numerous dose‐finding designs for drug combination have been proposed over the years. Copula‐type models exhibit distinctive advantages in this task over other models used in existing competitive designs. For example, their application enables the consideration of dose‐limiting toxicities attributable to one of two agents. However, if a particular combination therapy demonstrates extremely synergistic toxicity, copula‐type models are liable to induce biases in toxicity probability estimators due to the associated Fréchet–Hoeffding bounds. Consequently, the dose‐finding performance may be worse than those of other competitive designs. The objective of this study is to improve the performance of dose‐finding designs based on copula‐type models while maintaining their advantageous properties. We propose an extension of the parameter space of the interaction term in copula‐type models. This releases the Fréchet–Hoeffding bounds, making the estimation of toxicity probabilities more flexible. Numerical examples in various scenarios demonstrate that the performance (e.g., the percentage of correct MTDC selection) of the proposed method is better than those exhibited by existing copula‐type models and comparable with those of other competitive designs, irrespective of the existence of extreme synergistic toxicity. The results obtained in this study could motivate the real‐world application of the proposed method in cases requiring the utilization of the properties of copula‐type models.

## 1 INTRODUCTION

In recent anticancer therapies, treatment via combinations of anticancer drugs, such as cytotoxic agents, molecularly targeted agents, immune checkpoint inhibitor therapies, and effector cell therapies has become a routine practice. Each of these drugs exhibit unique mechanisms of action. Identification of the maximum tolerated dose combination (MTDC) of such combinations of anticancer drugs is one of the most important objectives in phase I oncology trials as toxicity is expected to increase with an increase in the doses of some of the constituent drugs (Simmet *et al.*, 2019). MTDC is defined as the dose combination that has a dose‐limiting toxicity (DLT) probability closest to the target toxicity probability.

Various types of dose‐finding designs have been proposed to identify MTDC accurately. For two‐drug combinations, Yin and Yuan (2009a) proposed a Bayesian dose‐finding design using the following copula‐type model based on the Clayton copula.

(1) $$\pi_{ij}=1-{\left\{{\left(1-p_{i}^{\alpha }\right)}^{-\gamma }+{\left(1-q_{j}^{\beta }\right)}^{-\gamma }-1\right\}}^{-1/\gamma }.$$

In the remainder of this study, (i,j) denotes the combination of the *i*th dose (i=1,2,…,I) of drug *A* and the *j*th dose (j=1,2,…,J) of drug *B*. Further, $\pi_{ij}$ represents the joint toxicity probability corresponding to dose combination (i,j). $p_{i}$ and $q_{j}$ are the prespecified toxicity probabilities corresponding to the *i*th dose of drug *A* and the *j*th dose of drug *B*, respectively, α>0,β>0, and γ>0 are the model parameters, and γ represents the effect of the drug–drug interaction. Yin and Yuan (2009a, 2009b) proposed two other copula‐type models based on the Gumbel–Murtaugh and Gumbel–Hougaard copulas.

The copula‐type model has distinctive properties that have been applied to dose‐finding designs considering specific situations. For example, Jimenez *et al.* (2019) proposed a design based on a copula‐type model for a trial in which a clinician is able to identify certain toxicities attributable to one of the two constituent agents. In addition, Wheeler *et al.* (2017) proposed a design applying a copula‐type model to a trial in which one of the two constituent agents is administered at the beginning of the treatment cycle and the other is administered at a later instant during the cycle. This allows DLTs observed before the administration of the second drug to be attributed to the first drug. The aforementioned examples instantiate the enhanced versatility of copula‐type models compared with models used in other competitive designs. However, Gasparini *et al.* (2010) argued that copula‐type models are not suitable for the estimation of joint toxicity probabilities in cases where extreme synergistic or extreme antagonistic behavior is expected, owing to the Fréchet–Hoeffding (F–H) bounds; $\max\left(p_{i},q_{j}\right)\leq \pi_{ij}\leq \min\left(p_{i}+q_{j},1\right)$. In response to Gasparini's comment, Yin and Yuan explained the nonexistence of lower and upper bounds in their model by arguing that the upper and lower bounds in their copula‐type model is given by

(2) $$\max\left(p_{i}^{\alpha },q_{j}^{\beta }\right)\leq \pi_{ij}\leq \min\left(p_{i}^{\alpha }+q_{j}^{\beta },1\right),$$

which allows $\pi_{ij}$ to take any value in the range between 0 and 1 (Yin and Yuan, 2010). However, this is valid only during the estimation of the joint toxicity probability corresponding to a single dose combination. As an illustrative example, let us consider the case in which the joint toxicity probabilities, $\pi_{12},\pi_{21}$, and π_22_, are equal to 0.05, 0.05, and 0.30, respectively. It is evident from Equation (2) that the maxima of π_12_ and π_21_ are $p_{1}^{\alpha }+q_{2}^{\beta }$ and $p_{2}^{\alpha }$ + $q_{1}^{\beta }$, respectively. They can be transformed into $p_{2}^{\alpha }=\pi_{21}-q_{1}^{\beta }$ and $q_{2}^{\beta }=\pi_{12}-p_{1}^{\alpha }$, respectively. Similarly, as the maximum of π_22_ is $p_{2}^{\alpha }$ + $q_{2}^{\beta },\pi_{22}=\pi_{21}-q_{1}^{\beta }+\pi_{12}-p_{1}^{\alpha }$ holds. It can be readily verified that, when $p_{1}^{\alpha }=q_{1}^{\beta }=0,\pi_{22}$ attains the maximum value. If π_21_ and π_12_ were estimated without the bias (i.e., 0.05), the bias of the estimator for π_22_ would be 0.20. On the contrary, the proximity of the estimated value of π_22_ to 0.30 is proportional to the bias of the estimator of π_21_ and π_12_. Therefore, irrespective of the values of α and β, a copula‐type model is restricted by the F–H bounds and the inequality, ${\hat{\pi }}_{i,j}<{\hat{\pi }}_{i-1,j}$ + ${\hat{\pi }}_{i,j-1}$, always holds in such models.

The degraded performance of dose‐finding designs based on copula‐type models compared with other competitive designs under scenarios involving extreme synergistic toxicity have also been verified in several studies (Wages *et al.*, 2011; Riviere *et al.*, 2014; Hirakawa *et al.*, 2015; Mander and Sweeting, 2015; Lin and Yin, 2016, 2017). Certain phase I trial reports have suggested that combination therapy demonstrates extreme synergistic toxicity. For example, 62 patients were evaluated in the phase Ib dose‐escalation study of the combination of the oral Pan‐PI3K inhibitor, buparlisib, and the oral MEK1/2 inhibitor, trametinib (Bedard *et al.*, 2015). Let (i,j) denote the dose levels of combinations of buparlisib and trametinib. The toxicity probabilities corresponding to dose combinations (1, 3), (1, 4), (2, 3), and (2, 4) were estimated to be 0.03, 0.06, 0.15, and 0.33, respectively, using a logistic regression model (Lam *et al.*, 2019). This suggests the existence of an extreme synergistic toxicity. In addition, extreme synergistic toxicity has been observed in the phase I study of the combination of neratinib and temsirolimus (Gandhi *et al.*, 2014). Therefore, dose‐finding design based on copula‐type models may not be suitable in several practical clinical trials.

The objective of this study is to improve the performances of dose‐finding designs based on copula‐type models while maintaining their advantageous properties. We propose an extension of the parameter space of γ in copula‐type models under the assumption that any function that takes values between 0 and 1 can be used to estimate $\pi_{ij}$. The proposed extension releases the F–H bounds, making the estimation of toxicity probabilities more flexible. We conduct numerical evaluations to verify the performance of the proposed method and compare it with those of other competitive designs.

The remainder of this paper is structured as follows. In Section 2, we describe the characteristics of a copula‐type model and introduce certain copula‐type models based on well‐known copulas. In addition, we formulate the proposed method. Section 3 details the simulation setting along with the dose‐escalation algorithm and MTDC‐determination procedure of each dose‐finding design. In Section 4, we explain the results of the numerical studies under various scenarios. Section 5 presents some concluding remarks and considerations.

## 2 PROBABILITY MODELS

### 2.1 Copula‐type models

Yin and Yuan (2009a, 2009b) introduced the categorization of the toxicity probability, $\pi_{ij}$, of the dose combination, (i,j), into the following four classes of joint probabilities.

(1) $\pi_{ij}^{11}$, associated with the DLT attributed to both drugs;
(2) $\pi_{ij}^{10}$, associated with the DLT attributed to drug *A*, but not to drug B;
(3) $\pi_{ij}^{01}$, associated with the DLT attributed to drug *B*, but not to drug *A*; and
(4) $\pi_{ij}^{00}$, which is the probability of not observing a DLT (i.e., the DLT attributed to drug A or drug B or both is not observed).

Owing to the difficulty of explicit attribution of an observed DLT to drug *A*, drug *B*, or both, the authors focused on $\pi_{ij}^{00}$ and proposed the estimation of $\pi_{ij}$ based on the transformation, $\pi_{ij}=1-\pi_{ij}^{00}$. Let C(·,·) denote a copula connecting univariate marginal distribution functions. The joint toxicity probability, $\pi_{ij}$, of the dose combination, (i,j), based on a copula‐type model is given by

$$\pi_{ij}=1-\pi_{ij}^{00}=1-C\left(1-F_{A}\left(p_{i}\right),1-F_{B}\left(q_{j}\right)\right).$$

Here, $F_{A}\left(p_{i}\right)=p_{i}^{\alpha }$ and $F_{B}\left(q_{j}\right)=q_{j}^{\beta }$ are the marginal cumulative distribution functions of the observed toxicity. $p_{i}$ and $q_{j}$ represent the prespecified toxicity probabilities corresponding to the *i*th dose of drug *A* and the *j*th dose of drug *B*, respectively, where $p_{1}<p_{2}<\text{…}\;<p_{I}$ and $q_{1}<q_{2}<\text{…}\;<q_{J}$. The Clayton copula‐type model given by Equation (1) is derived by the Clayton copula of $C\left(x,y\right)={\left(x^{-\gamma }+y^{-\gamma }-1\right)}^{-1/\gamma }$. Other copulas may also be applied in a similar fashion. Yin and Lin (2015) compared the performances of dose‐finding designs based on six types of copula‐type models and demonstrated that there is no substantial difference between them.

Table 1 illustrates the copula‐type models based on well‐known one‐parameter copulas (two‐dimensional copulas) along with the respective parameter spaces of γ and the ranges of $\pi_{ij}$ under fixed values of $p_{i}^{\alpha }=q_{j}^{\beta }=0.15$. As is evident from the table, the parameter space of γ and the range of $\pi_{ij}$ are unique for each model. The Clayton and Frank model attains the upper and lower F–H bounds given by Equation (2). However, even in the case of the Clayton and Frank model, the range of $\pi_{ij}$ is not sufficiently wide. This is because, in cases involving extreme synergistic toxicity, $\pi_{ij}$ is expected to be greater than min($p_{i}^{\alpha }+q_{j}^{\beta }$, 1). Thus, in such cases, dose‐finding designs based on copula‐type models are not effective. In contrast, copula‐type models exhibit the following properties:

(1) if $p_{i}^{\alpha }$ = 0 and $q_{j}^{\beta }$ = 0, then $\pi_{ij}$ = 0;
(2) if $p_{i}^{\alpha }$ = 0, then $\pi_{ij}$ = $q_{j}^{\beta }$, and if $q_{j}^{\beta }$ = 0, then $\pi_{ij}$ = $p_{i}^{\alpha }$;
(3) if $p_{i}^{\alpha }$ = 1 or $q_{j}^{\beta }$ = 1, then $\pi_{ij}$ = 1;
(4) there is a unique value of γ for which $\pi_{ij}$ =1 $-\left(1-p_{i}^{\alpha }\right)(1-q_{j}^{\beta }$).

Property (2) is notable because it asserts that $\pi_{ij}$ is equal to the toxicity probability of one drug if the toxicity probability of the other drug within the combination is known to be zero. As discussed in Section 1, several dose‐finding designs have been proposed based on this property and the concepts of copula‐type models described at the beginning of this section (Wheeler *et al.*, 2017; Jimenez *et al.*, 2019). Property (4) implies that any copula‐type model is capable of expressing $\pi_{ij}$ with no interaction. In addition to a copula‐type model, the models proposed by Thall *et al.* (2003) and Wang and Ivanova (2005) also satisfy the properties of (1) to (3).

**TABLE 1:** Various copula‐type models

Copula^a^	Copula‐type models at dose combination, (i,j)	Parameter space of γ	Range of $\pi_{ij}$ at $p_{i}^{\alpha }=q_{j}^{\beta }=0.15$
Clayton	$\pi_{ij}=1-{\left\{{\left(1-p_{i}^{\alpha }\right)}^{-\gamma }+{\left(1-q_{j}^{\beta }\right)}^{-\gamma }-1\right\}}^{-1/\gamma }$	[−1,∞)\{0}^b^	[0.15, 0.30]
GUMM	$\pi_{ij}=1-(1-p_{i}^{\alpha })(1-q_{j}^{\beta })\left(1+p_{i}^{\alpha }q_{j}^{\beta }\left(\frac{e^{\gamma }-1}{e^{\gamma }+1}\right)\right)$	(−∞,∞)	[0.26, 0.29]
GUMH	$\pi_{ij}=1-\exp[-{\left\{{\left(-\log(1-p_{i}^{\alpha })\right)}^{\gamma }+{\left(-\log(1-q_{j}^{\beta })\right)}^{\gamma }\right\}}^{1/\gamma }]$	[1,∞)	[0.15, 0.28]
Frank	$\pi_{ij}=1+\frac{1}{\gamma }\log\left\{1+\frac{\left(e^{-\gamma (1-p_{i}^{a})}-1\right)(e^{-\gamma (1-q_{j}^{B})}-1)}{e^{-\gamma }-1}\right\}$	(−∞,∞)\{0}	[0.15, 0.30]
AMH	$\pi_{ij}=1-(1-p_{i}^{\alpha })(1-q_{j}^{\beta })/(1-\gamma p_{i}^{\alpha }q_{j}^{\beta })$	[−1,1)	[0.26, 0.29]
Joe	$\pi_{ij}={\left(p_{i}^{\alpha \gamma }+q_{j}^{\beta \gamma }-p_{i}^{\alpha \gamma }q_{j}^{\beta \gamma }\right)}^{1/\gamma }$	[1,∞)	[0.15, 0.28]
FGM	$\pi_{ij}=1-(1-p_{i}^{\alpha })(1-q_{j}^{\beta })(1+\gamma p_{i}^{\alpha }q_{j}^{\beta })$	[−1,1)	[0.26, 0.29]

^*a*^GUMM: Gumbel‐Murtaugh, GUMH: Gumbel‐Hougaard, AMH: Ali‐Mikhail‐Haq, FGM: Farlie‐Gumbel‐Morgenstern

^*b*^Yin and Yuan (2009a) defined γ>0, but in practice, the range −1<γ<∞ is used.

In general, the parameter space of γ corresponding to each model is derived to ensure that the two‐dimensional joint distribution is an actual probability distribution. We outline the derivation of the parameter space of the Ali‐Mikhail‐Haq (AMH) model (Ali *et al.*, 1978). The two‐dimensional joint distribution function (JDF), $F(p_{i},q_{j})$, connected by the AMH copula, which corresponds to the joint toxicity probability, $\pi_{ij}^{11}$, is given by

$$F\left(p_{i},q_{j}\right)=\frac{F_{A}\left(p_{i}\right)F_{B}\left(q_{j}\right)}{1-\gamma \left(1-F_{A}\left(p_{i}\right)\right)\left(1-F_{B}\left(q_{j}\right)\right)}.$$

In addition, the corresponding joint toxicity probability density function is given by

$$f\left(p_{i},q_{j}\right)=\frac{\partial^{2}F\left(p_{i},q_{j}\right)}{\partial p_{i}\partial q_{j}}={\left(F\left(p_{i},q_{j}\right)\right)}^{2}\frac{1-\gamma +2\gamma F\left(p_{i},q_{j}\right)}{{\left(F_{A}\left(p_{i}\right)F_{B}\left(q_{j}\right)\right)}^{2}}.$$

It is readily apparent that $0\leq F_{A}\left(p_{i}\right),F_{B}\left(q_{j}\right)\leq 1,F\left(p_{i},1\right)=F_{A}\left(p_{i}\right),F\left(1,q_{j}\right)=F_{B}\left(q_{j}\right),F\left(p_{i},0\right)=F\left(0,q_{j}\right)=0$, and F(1,1)=1. To ensure that $F(p_{i},q_{j})\geq 0$ for all $p_{i}$ and $q_{j},\gamma$ is required to satisfy γ<1. In addition, to ensure that the joint toxicity probability density function, $f(p_{i},q_{j})\geq 0$ for all $p_{i}$ and $q_{j},\gamma$ is required to satisfy −1≤γ<1. Therefore, by combining the constraints introduced by the desired properties of $f(p_{i},q_{j})$ and $F(p_{i},q_{j})$, the parameter space of γ is identified to be −1≤γ<1.

### 2.2 Flexibility of application of copula‐type models

In copula‐type models, the likelihood is simply a product of the binomial probability mass function, which is given by

(3) Lα,β,γ|D=∏i=1I∏j=1Jnijmij1−πijnij−mijπijmij,

where *D* denotes the data collected until a given instant, and $n_{ij}$ and $m_{ij}$ denote the number of patients who received the drug combination, (i,j), and experienced DLTs corresponding to the drug combination, (i,j), respectively. We assume the DLT outcomes of all patients to be independent. In a copula‐type model, $\pi_{ij}$ is represented by a two‐dimensional JDF connecting two univariate marginal distribution functions. The parameter space of γ in a copula‐type model is determined such that it meets the two conditions mentioned in Section 2.1. By relaxing one of these conditions, $\partial^{2}F\left(p_{i},q_{j}\right)$/$\partial p_{i}\partial q_{j}\geq 0$, we propose an extension of the parameter space of γ within the range satisfying the restriction that $\pi_{ij}$ should lie within 0 and 1, thereby corresponding to the condition $0\leq F(p_{i},q_{j})\leq 1$. This extension of γ leads to an increase in the F–H upper bound (henceforth denoted by $\pi_{ij}^{\mathrm{upper}}$). The proposed method with this extended parameter space utilizes the regression model using the same formula (function) as the original copula‐type model to estimate $\pi_{ij}$. The function in the original method corresponds to a JDF derived using a copula, but the function in the proposed method does not completely satisfy the condition of a JDF because the corresponding density function, $f\left(p_{i},q_{j}\right)=\partial^{2}F\left(p_{i},q_{j}\right)/\partial p_{i}\partial q_{j}$, does not always provide a positive value in the extended range of γ. Here, $F\left(p_{i},q_{j}\right)=C\left(1-p_{i},1-q_{j}\right)$, where *C* represents a copula. The function in the proposed method is not a JDF but a mere function that takes values between 0 and 1; however, the proposed method inherits the advantageous properties of copula‐type models. Web Appendix A records the properties of a copula that are preserved by the proposed method, along with the proofs of these statements, and establishes that the proposed method is equipped with the minimal properties of a copula that are required for the modeling of $\pi_{ij}$. Here, we would like to emphasize that any function that takes values between 0 and 1 can be used for modeling the relationship between the DLTs and dose combinations because $\pi_{ij}$ is only a parameter of the binomial probability mass function in the likelihood of Equation (3). When $0\leq \pi_{ij}\leq 1$ is preserved, the resultant likelihood is proper.

Table 2 lists the extended parameter spaces of γ and the corresponding ranges of $\pi_{ij}$. We selected the AMH, GUMH (Gumbel‐Hougaard, AMH: Ali‐Mikhail‐Haq), and Joe models because their parameter spaces of γ can be extended easily irrespective of $p_{i}^{\alpha }$ and $q_{j}^{\beta }$. For instance, in the case of AMH, the parameter space of γ is (−∞, 1). However, in the case of FGM (Farlie‐Gumbel‐Morgenstern), it is required to be above $-{\left(p_{i}^{\alpha }q_{j}^{\beta }\right)}^{-1}$ to ensure that $\pi_{ij}$ lies between 0 and 1. It is evident from Table 2 that the extension of the parameter space of γ enables $\pi_{ij}^{\mathrm{upper}}$ to be increased to 1. The model proposed by Wang and Ivanova (2005) was not developed as a copula‐type model, but Gasparini (2013) used his concept of dose‐free parameterization to prove that it is equivalent to a copula‐type model if the original parameter space of 0<γ is restricted to 0 <γ< 1. If the model is not regarded as a copula‐type model, it seems natural not to restrict the parameter space, as done by Wang and Ivanova. It appears that their model with its original parameter space is one that can be constructed based on the proposed method.

**TABLE 2:** Extended parameter space of γ and ranges of $\pi_{ij}$

Copula	Type	Parameter space of γ	Range of $\pi_{ij}$ at $p_{i}^{\alpha }=q_{j}^{\beta }=0.15$
AMH	Original	[−1,1)	[0.26, 0.29]
	Extension	(−∞,1)	[0.26, 1.00]
GUMH	Original	[1,∞)	[0.15, 0.28]
	Extension	(0,∞)	[0.15, 1.00]
Joe	Original	[1,∞)	[0.15, 0.28]
	Extension	(0,∞)	[0.15, 1.00]

The stopping rule used in original copula‐type models can be implemented in the proposed method as well. The trial can be terminated at an early stage for safety if even the lowest dose combination is overly toxic, as indicated by Pr($\pi_{11}>\phi |$ D) >λ, where λ denotes a threshold value close to 1.

## 3 SIMULATION SETTINGS

### 3.1 Common settings

We conducted simulation studies under the 18 scenarios listed in Table 3 to investigate the operating characteristics of a dose‐finding design based on an existing copula‐type model (COPULA), the proposed method based on the extension of the parameter space of γ (COPULA‐E), and four existing dose‐finding designs—two‐dimensional model‐based design (TDM) (Wang and Ivanova, 2005), logistic model‐based design (LOGI) (Riviere *et al.*, 2014), partial ordering CRM design (POCRM) (Wages *et al.*, 2011), and two‐dimensional Bayesian optimal interval design (BOIN) (Lin and Yin, 2017). We utilized three copula‐type models, namely, AMH, GUMH, and Joe in both COPULA (the designs are denoted by AMH‐O, GUMH‐O, and Joe‐O) and COPULA‐E (the designs are denoted by AMH‐E, GUMH‐E, and Joe‐E). The 18 scenarios comprised the 16 scenarios considered by Hirakawa *et al.* (2015) and 2 additional scenarios that do not involve synergistic toxicity corresponding to any of the dose combinations. Of the 18 scenarios, we considered scenarios 4, 5, 6, 7, 8, 14, 15, and 16 to exhibit extreme synergistic toxicity around true MTDCs. The scenario satisfying the condition, Pr(i,j)> {Pr(i−1,j)− Pr(i−1,j−1)} + {Pr(i,j−1)− Pr(i−1,j−1)} + Pr(i−1,j−1), was identified to be the scenario exhibiting extreme synergistic toxicity, where Pr(x,y) represents the toxicity probability of the dose combination, (x,y), and the dose combination, (i,j), is an MTDC.

**TABLE 3:** Trials of combinations of agents under 18 scenarios (MTDCs are highlighted in boldface)

A											
		1	2	3	4	5	1	2	3	4	5
			Scenario 1					Scenario 2			
	3	**0.30**	0.40	0.50			0.50	0.70	0.80		
	2	0.20	**0.30**	0.40			**0.30**	0.60	0.70		
	1	0.10	0.20	**0.30**			0.05	**0.30**	0.50		
			Scenario 3					Scenario 4			
	3	0.40	0.60	0.80			0.15	0.40	0.60		
	2	**0.30**	0.50	0.70			0.05	**0.30**	0.40		
	1	0.05	0.10	0.40			0.01	0.05	0.15		
			Scenario 5					Scenario 6			
	4	**0.30**	0.50	0.65	0.70		**0.30**	0.50	0.60	0.70	
	3	0.10	**0.30**	0.60	0.65		0.15	0.40	0.50	0.60	
	2	0.05	0.10	**0.30**	0.50		0.10	**0.30**	0.40	0.50	
	1	0.01	0.05	0.10	**0.30**		0.05	0.10	0.15	**0.30**	
			Scenario 7					Scenario 8			
	4	0.40	0.45	0.60	0.85		0.15	0.60	0.75	0.80	
	3	0.15	**0.30**	0.55	0.60		0.10	0.45	0.70	0.75	
	2	0.08	0.15	0.23	**0.30**		0.04	**0.30**	0.45	0.60	
	1	0.01	0.02	0.03	0.04		0.02	0.10	0.15	0.40	
B											
			Scenario 9					Scenario 10			
	2	0.10	0.20	**0.30**	0.40		**0.30**	0.40	0.50	0.60	
	1	0.05	0.10	0.20	**0.30**		0.01	0.10	0.20	**0.30**	
			Scenario 11					Scenario 12			
	2	0.15	**0.28**	**0.32**	**0.34**		0.50	0.60	0.70	0.80	
	1	0.10	0.15	**0.28**	**0.32**		0.10	0.20	**0.30**	0.40	
			Scenario 13					Scenario 14			
	3	0.25	**0.30**	0.40	0.50	0.70	0.20	0.45	0.50	0.60	0.75
	2	0.10	0.25	**0.30**	0.40	0.50	0.05	**0.30**	0.45	0.55	0.60
	1	0.05	0.10	0.25	**0.30**	0.40	0.01	0.05	0.15	**0.30**	0.50
			Scenario 15					Scenario 16			
	3	**0.30**	0.35	0.40	0.45	0.60	0.10	0.20	0.40	0.55	0.65
	2	0.05	0.20	**0.30**	0.40	0.45	0.05	0.10	**0.30**	0.50	0.60
	1	0.01	0.05	0.10	0.20	**0.30**	0.01	0.05	0.10	0.20	0.40
			Scenario 17					Scenario 18			
	3	**0.31**	0.38	0.51			**0.31**	0.37	0.41	0.51	0.58
	2	0.12	0.21	0.37			0.11	0.19	0.24	0.37	0.46
	1	0.04	0.14	**0.31**			0.02	0.11	0.16	**0.31**	0.41

In all of the scenarios considered, the target toxicity probability, ϕ, was set to 0.3, and dose combinations with toxicity probabilities greater than or equal to 0.35 were defined to be overdose combinations (OCs). The total sample size was taken to be 30 with a cohort size of 3. A total of 1000 trials were simulated corresponding to each scenario. We did not employ any stopping rules, that is, the MTDC is determined after attaining the maximum sample size.

We evaluated the operating characteristics in terms of six outcome metrics. The first three outcome metrics are the percentage of correct MTDC selections, percentage of OC selections, and accuracy index (AI) proposed by Cheung (2011); these represent the efficiency and accuracy of a dose‐finding design. The remaining three outcome metrics are the overall percentage of observed toxicity, percentage of patients allocated to MTDC, and percentage of patients allocated to OC; these represent the safety perspectives of a trial. AI is defined as follows.

$$AI=1-I\times J\times \frac{\sum_{i=1}^{I}\sum_{j=1}^{J}\left|\pi_{ij}^{*}-\phi \right|\times w_{ij}}{\sum_{i=1}^{I}\sum_{j=1}^{J}\left|\pi_{ij}^{*}-\phi \right|},$$

where $\pi_{ij}^{*}$ indicates the true toxicity probability of the dose combination, (i,j), and $w_{ij}$ denotes the probability of selecting the dose combination, (i,j), as the MTDC. The maximum value of AI is 1, and larger values correspond to higher dose‐finding accuracies. In this study, we multiplied all values of AI by 100.

The model parameters for COPULA, COPULA‐E, LOGI, and TDM are estimated via the MCMC using PROC MCMC of SAS 9.4 (SAS Institute, Cary, NC). We record 10 000 posterior samples after 1000 burn‐in iterations. In addition, the number of chains and the thinning interval are taken to be 1 and 3, respectively.

### 3.2 Settings of each dose‐finding design

In COPULA and COPULA‐E, we replace α and β in the models shown in Table 1 with exp(α) and exp(β), respectively. We assume the prior distribution of α and β to be standard normal for all copula‐type models of both COPULA and COPULA‐E. In COPULA, the prior distribution of γ is assumed to be uniform (−1, 1) in the AMH model and truncated $N{\left(1,3^{2}\right)}_{\left([1,\infty ]\right)}$ in the GUMH and Joe models. In COPULA‐E, the prior distribution of γ is assumed to be truncated N(0, ${1500^{2})}_{\left[-\infty ,1\right)}$ in the AMH model and Gamma(0.5, 0.5) in the GUMH and Joe models, respectively. The initial estimates of the toxicity probability, $p_{i}$, are set to $\left(p_{1},p_{2}\right)=\left(0.1,0.3\right)$ for $I=2,\left(p_{1},p_{2},p_{3}\right)=\left(0.1,0.2,0.3\right)$ for *I* = 3, $\left(p_{1},p_{2},p_{3},p_{4}\right)=\left(0.075,0.15,0.225,0.3\right)$ for I=4, and $\left(p_{1},p_{2},p_{3},p_{4},p_{5}\right)=\left(0.06,0.12,0.18,0.24,0.30\right)$ for *I* = 5, respectively. The same settings are used for the initial estimates of the toxicity probability, $q_{j}$. The dose‐toxicity model in TDM is taken to be $\pi_{ij}=1-{\left(1-p_{i}\right)}^{\alpha }{\left(1-q_{j}\right)}^{\left\{\beta +\gamma \log\left(1-p_{i}\right)\right\}}$, where α>0,β>0, and γ<0. The prior distribution of α,β, and γ are assumed to be Gamma(1, 1). The LOGI model is given by $\pi_{ij}=\beta_{0}+\beta_{1}u_{i}+\beta_{2}v_{j}+\beta_{3}u_{i}v_{j}$, where $u_{i}\;=\;\log\left\{p_{i}/\left(1-p_{i}\right)\right\},v_{j}\;=\;\log\left\{q_{j}/\left(1-q_{j}\right)\right\},-\infty \;<\;\beta_{0}\;<\;\infty ,\beta_{1}>0,\beta_{2}>0,\forall j\;\beta_{1}+\beta_{3}v_{j}>0$, and $\forall i\;\beta_{2}+\beta_{3}u_{i}>0$. The prior distributions of β_0_ and β_3_ are assumed to be N(0,10), and those of β_1_ and β_2_ are assumed to be Gamma(1,1). The values of $p_{i}$ and $q_{j}$ in COPULA are used without alteration as the initial estimates for TDM and LOGI. In the case of POCRM, skeleton values (the initial estimates) are obtained in the R package, dfcrm. In particular, we use the functions getprior(0.05, 0.30, 4, 9) for 3× 3 combinations, getprior(0.05, 0.30, 7, 16) for 4× 4 combinations, getprior(0.05, 0.30, 4, 8) for 2× 4 combinations, and getprior(0.05, 0.30, 7, 15) for 3 × 5 combinations. Following the details of POCRM introduced by Wages and Conaway (2013), we select eight possible orders with equal prior probabilities. We obtain the results of BOIN by using the application available online under their default settings.

### 3.3 Dose‐escalation algorithm and MTDC determination

In all of the dose‐finding designs considered, the first cohort is treated at the lowest dose combination (1, 1). Following is the description of the dose combination for the next cohort. In the case of BOIN, the dose‐escalation algorithm proposed in the original article is applied. The dose‐escalation algorithms of other designs comprise two stages. During the first stage, the dose is escalated based on a prespecified rule without using statistical models. This is continued until one DLT is observed (alternatively, in the case of POCRM, until the maximum likelihood estimates are obtained). During the second stage, the dose is escalated or de‐escalated based on the values predicted by the models. Although the dose‐finding designs usually utilize distinct first stages, the first stage proposed by Wages *et al.* (2011) is employed for all designs in this study, to ensure homogeneity of conditions for a fair comparison. During this stage, the dose is sequentially escalated from the lowest dose group to the highest dose group. A dose group is defined to be a set of dose combinations lying on the same diagonal in the drug combination matrix. Two drug combinations lie on the same diagonal if the sums of their corresponding dose levels are equal. When multiple dose combinations exist in a dose group, one of them is sampled without replacement for the next cohort. This sampling is continued until all dose combinations in a dose group are selected.

During the second stage, the dose‐escalation algorithm proposed in each original article is applied to the corresponding dose‐finding design. With respect to the current dose combination, (i,j), the second stage of COPULA and COPULA‐E can be described as follows.

- If Pr$(\pi_{ij}<\phi |D)>ce$, the current dose combination is escalated to the adjacent dose combination, (i+1,j),(i,j+1),(i+1,j−1),(i−1,j+1), whose toxicity probability is closest to ϕ. If the current dose combination is (I,J), which is the highest dose combination, we retain it for the next cohort.
- If Pr$(\pi_{ij}>\phi |D)>cd$, the current dose combination is de‐escalated to the adjacent dose combination, (i−1,j),(i,j−1),(i+1,j−1),(i−1,j+1), whose toxicity probability is closest to ϕ. If the current dose combination is (1, 1), which is the lowest dose combination, we retain it for the next cohort.
- If Pr$(\pi_{ij}<\phi |D)\leq ce$ and Pr$(\pi_{ij}>\phi |D)\leq cd$, we retain the current dose combination for the next cohort,

where ce and cd represent the threshold for dose escalation and dose de‐escalation, respectively, satisfying ce+cd>1. We take ce = 0.75 and cd = 0.55 for COPULA and COPULA‐E. LOGI employs the same algorithm as COPULA, and we use their default settings—ce = 0.85 and cd = 0.55—in its case.

The MTDC‐determination procedure proposed in each original article is applied to the corresponding dose‐finding design. COPULA and COPULA‐E are taken to employ the determination method used in LOGI. In LOGI, the MTDC is selected from the dose combinations used to treat patients and the dose combination with the highest posterior probability, Pr$\left(\pi_{ij}\in \left[\phi -\delta ,\phi +\delta \right]|D\right)$, is taken to be the MTDC, where we set δ=0.1.

## 4 RESULTS

### 4.1 COPULA versus COPULA‐E (proposed method)

In this subsection, we compare the performances of the original method (COPULA: AMH‐O, GUMH‐O, and Joe‐O) and the proposed method (COPULA‐E: AMH‐E, GUMH‐E and Joe‐E). Table 4 presents the measured values of the first three outcome metrics for the efficiency of a dose‐finding design. The three outcome metrics for the safety aspects are depicted in Web Table 2 in Web Appendix B.

(1) Average performance:    Across all the 18 scenarios, the MTDC selection percentages were observed to be, respectively, 34% and 44% for AMH‐O and AMH‐E (10% improvement); 32% and 41% for GUMH‐O and GUMH‐E (9% improvement); and 33% and 38% for Joe‐O and Joe‐E (5% improvement). Moreover, AMH‐E, GUMH‐E, and Joe‐E exhibited OC selection percentages that were 16%, 14%, and 7% smaller than those exhibited by AMH‐O, GUMH‐O, and Joe‐O, respectively. AMH‐E, GUMH‐E, and Joe‐E exhibited improvements by 17, 18, and 10 in AI compared to AMH‐O, GUMH‐O, and Joe‐O, respectively. Overall, AMH‐E performed the best with respect to the majority of outcome metrics, including the percentage of correct MTDC selection.
(2) Performance of each scenario:    AMH‐E improved the MTDC selection percentage by more than 10% compared to AMH‐O in scenarios 2, 4, 5, 6, 8, 12, 14, 15, 16, and 17. There was no scenario in which AMH‐E was inferior to AMH‐O by greater than 4% in terms of this metric. The OC selection percentage achieved by AMH‐E was lower than that of AMH‐O by more than 10% in all scenarios except 2, 3, and 11. GUMH‐E also improved the MTDC selection percentage by more than 10% compared to GUMH‐O in scenarios 2, 4, 5, 6, 8, 10, 12, and 15. The OC selection percentage achieved by GUMH‐E was less than that of GUMH‐O by at least 10% in all scenarios except 2, 3, 11, and 12. Joe‐E improved the MTDC selection percentage by more than 10% compared to Joe‐O in scenarios 5, 6, 8, 10, and 15. The OC selection percentage achieved by Joe‐E was less than that of Joe‐O by at least 10% in scenarios 9, 10, 13, 15, 16, and 18. More importantly, in all scenarios suggesting the presence of extreme synergistic toxicity, AMH‐E and GUMH‐E exhibited substantial improvements over AMH‐O and GUMH‐O, respectively. Furthermore, COPULA‐E exhibited better MTDC selection percentages corresponding to all three copula‐type models even in scenarios 17 and 18, which involved no synergistic toxicity.

**TABLE 4:** Values of the three outcome metrics for COPULA vs. COPULA‐E. Scenarios highlighted in gray represent scenarios involving extreme synergistic toxicity. Avg represents the average across the 18 scenarios

	Scenarios																		
Designs	1	2	3	4	5	6	7	8	9	10	11	12	13	14	15	16	17	18	Avg
	MTDC selections (%)																		
AMH‐O	42	74	38	24	43	28	27	17	38	44	79	22	35	31	21	5	24	18	34
AMH‐E	48	84	34	39	66	48	33	33	39	53	77	39	36	41	38	22	37	22	44
GUMH‐O	39	70	38	26	32	24	26	18	39	36	84	24	31	27	15	5	21	15	32
GUMH‐E	44	82	44	39	58	44	31	32	41	51	82	34	35	31	34	13	28	22	41
Joe‐O	41	69	40	25	36	24	26	16	37	39	87	25	33	29	16	4	22	19	33
Joe‐E	43	78	46	26	50	39	30	26	41	55	77	23	35	26	27	11	25	23	38
	OC selections (%)																		
AMH‐O	28	15	42	66	43	51	54	55	34	37	‐	32	34	54	40	65	45	53	44
AMH‐E	15	13	48	41	20	26	31	39	15	25	‐	20	20	33	19	41	29	35	28
GUMH‐O	30	15	42	66	52	57	57	54	38	48	‐	32	39	57	46	67	51	58	48
GUMH‐E	20	12	38	47	31	39	43	44	22	31	‐	23	22	40	25	49	39	44	34
Joe‐O	31	16	40	66	47	53	54	57	39	45	‐	30	35	55	47	71	49	56	46
Joe‐E	22	13	41	59	41	46	50	51	26	31	‐	31	24	52	34	60	42	46	39
	Accuracy index																		
AMH‐O	26	73	50	35	32	33	33	37	43	50	33	50	39	38	37	22	24	28	38
AMH‐E	36	86	60	57	64	57	57	59	44	64	32	67	57	58	59	47	39	40	55
GUMH‐O	18	69	47	31	15	25	25	32	46	39	41	50	35	33	25	16	19	26	33
GUMH‐E	30	83	61	56	54	53	50	57	46	60	39	60	54	48	54	40	33	38	51
Joe‐O	20	68	49	30	21	25	28	34	44	42	45	51	40	34	26	13	23	29	35
Joe‐E	31	78	58	41	42	47	44	47	45	62	30	51	51	41	46	29	30	37	45

### 4.2 COPULA‐E (Proposed method) vs. existing designs

In this subsection, we compare the performances of four existing designs (LOGI, TDM, POCRM, and BOIN) with that of AMH‐E, which exhibited the best performance among the variants of COPULA‐E based on the three copula‐type models. Table 5 shows the values of the first three outcome metrics for the design efficiency. The measured values of the three outcome metrics for the safety aspects are included in Web Table 3 in Web Appendix B.

(1) Average performance: Across all 18 scenarios, all dose‐finding designs except TDM exhibited similar performances, with MTDC selection percentages ranging between 43% and 45%. TDM performed the worst with a score of 34%. In addition, AMH‐E and LOGI exhibited the lowest percentage of OC selection at 28%, followed by POCRM (29%) and BOIN (30%) and, finally, TDM (42%). Similarly, TDM exhibited the lowest AI value of 36 and the other designs exhibited similar performance, with AIs ranging between 53 and 56. Although LOGI performed the best in terms of the first three outcome metrics on average across 18 scenarios, the differences between the performances of most of the methods were small.
(2) Performance of each scenario: The MTDC selection percentages of AMH‐E, LOGI, and BOIN were observed to be comparable in each scenario. However, BOIN exhibited an OC selection percentage that was 10% higher than those of AMH‐E and LOGI in scenarios 1, 12, 15, and 17. The MTDC selection percentage of POCRM was 10% lower than those of AMH‐E, LOGI, and BOIN in scenarios 2 and 4, even though it exhibited the highest MTDC selection percentages in scenarios 1, 9, 10, 13, and 16. TDM was observed to perform poorly in most scenarios in terms of both MTDC and OC selection percentages. The values of AI in each scenario were in agreement with both the MTDC and OC selection percentages. AMH‐E was not observed to be significantly inferior to any of the other dose‐finding designs in terms of any metric in any scenario.

**TABLE 5:** Values of the three outcome metrics for AMH‐E vs. existing competitive dose‐finding designs. Scenarios highlighted in gray denote the existence of extreme synergistic toxicity. Avg represents the average across the 18 scenarios

	Scenarios																		
Designs	1	2	3	4	5	6	7	8	9	10	11	12	13	14	15	16	17	18	Avg
	MTDC selections (%)																		
AMH‐E	48	84	34	39	66	48	33	33	39	53	77	39	36	41	38	22	37	22	44
LOGI	52	85	41	35	72	47	37	38	41	48	79	41	34	43	42	25	30	18	45
TDM	46	72	38	18	41	29	23	13	38	49	82	32	30	23	26	3	30	27	34
POCRM	58	69	32	20	69	45	35	33	48	62	78	33	43	37	36	27	31	20	43
BOIN	46	81	35	30	73	48	42	30	44	52	79	30	38	42	35	23	27	22	43
	OC selections (%)																		
AMH‐E	15	13	48	41	20	26	31	39	15	25	‐	20	20	33	19	41	29	35	28
LOGI	15	14	48	46	17	35	26	43	14	27	‐	19	15	39	17	39	30	34	28
TDM	30	19	44	61	44	50	56	54	36	34	‐	32	38	49	43	67	44	51	44
POCRM	18	29	58	48	18	30	22	37	16	29	‐	38	12	37	22	31	37	36	30
BOIN	27	17	50	42	16	33	25	39	23	30	‐	33	21	31	39	36	42	39	32
	Accuracy index																		
AMH‐E	36	86	60	57	64	57	57	59	44	64	32	67	57	58	59	47	39	40	55
LOGI	43	87	61	53	69	58	60	61	46	60	36	67	61	58	61	51	36	40	56
TDM	28	71	50	25	25	30	27	35	42	52	37	57	37	32	31	14	30	33	36
POCRM	51	73	57	41	65	55	52	56	48	65	34	63	68	52	53	48	43	42	54
BOIN	34	83	57	49	71	57	61	56	49	59	34	54	59	58	52	51	34	40	53

## 5 DISCUSSION AND CONCLUSION

The performance of the dose‐finding designs based on existing copula‐type models have been reported to be somewhat inferior to those of other dose‐finding designs (Wages *et al.*, 2011; Riviere *et al.*, 2014; Mander and Sweeting, 2015; Lin and Yin, 2016, 2017). Hirakawa *et al.* (2015) reported the MTDC selection percentage of the dose‐finding design using the Clayton copula‐type model to be 34% on average across 16 scenarios, which was approximately 10% lower than that of other dose‐finding designs. This is primarily attributed to the presence of extreme synergistic toxicity in the majority of scenarios considered by the authors. In particular, copula‐type models are incapable of accurately estimating joint toxicity probabilities in such scenarios due to F–H bounds, which reduce the MTDC selection percentages.

In this study, we proposed a relatively more flexible method to estimate toxicity probabilities by extending the parameter space of γ in copula‐type models. For dose‐finding designs based on the copula‐type models, AMH, GUMH, and Joe, we conducted numerical studies corresponding to 18 scenarios, including the 16 scenarios considered by Hirakawa *et al.* (2015). The simulation results revealed that the MTDC selection percentages were improved by approximately 10% by using the proposed method (AMH‐E and GUMH‐E) compared with the utilization of COPULA (AMH‐O and GUMH‐O) on average across 18 scenarios. Further, we verified that the performance of AMH‐E is comparable to those of three other existing dose‐finding designs (LOGI, POCRM, and BOIN) and superior to that of TDM. In addition, we evaluated the absolute performance of dose‐finding designs featured in the paper using the nonparametric optimal benchmark proposed by Mozgunov *et al.* (2021). Based on the benchmark assessment, the best methods are LOGI and AMH‐E, followed by POCRM, BOIN, GUMH‐E, and Joe‐E. The original methods, AMH‐O, GUMH‐O, and Joe‐O, and TDM, perform poorly compared to the other methods (see Web Appendix C).

In order to evaluate the applicability of the proposed method, further numerical studies were conducted. We verified that the stopping rule of the proposed method worked well under the 18 scenarios listed in Table 3 and four additional scenarios where all dose combinations are considered to be overly toxic (see Web Appendix D). Further, in the four scenarios where the toxicity probabilities of all dose combinations are extremely low, we verified that the proposed design escalates the dose to the highest dose combination as smoothly as the original design (see Web Appendix E).

For AMH‐E, we conducted a further numerical study by applying different standard deviation (SD) of 50, 100, 500, 1000, 1500, and 3000 to a prior distribution of γ. The corresponding results are presented in Web Table 10 in Web Appendix F. In ascending order of SD values, the average MTDC selection percentages and OC selection percentages over all 18 scenarios were 40%, 40%, 42%, 43%, 44%, 44%, and 34%, 33%, 30%, 29%, 28%, and 28%, respectively (SD = 1500 was applied in Section 3). It appears that AMH‐E with a prior distribution of γ assuming a larger drug–drug interaction exhibited better performance. The averaged posterior means of γ over the 18 scenarios were −38,−76,−369,−729,−1084, and −2144, respectively. Although γ=0 indicates no synergistic toxicity in the AMH copula, the posterior means were quite far from 0 even in scenarios 17 and 18, which involved no synergistic toxicity. The posterior mean was strongly influenced by the prior settings. We evaluated the bias of a toxicity probability estimator. The results indicate that the AMH‐E can estimate toxicity probabilities with less bias on average than the AMH‐O with or without the existence of extreme synergistic toxicity (see Appendix G). In addition, the larger the SD, the smaller the bias. However, if a prior distribution of γ with a larger SD is considered, the estimated marginal toxicity probabilities tend to be smaller. Given these insights, we recommend using a prior distribution of γ with a larger SD in AMH‐E (e.g., SD between 500 and 1500). However, if the estimation of the marginal toxicity probabilities is important, the following two solutions should be considered. The first is the use of smaller SDs for prior distributions of γ, although the design performance is expected to be somewhat reduced in this case. The second is to seek more appropriate sets of $\gamma ,p_{i}^{\alpha }$ and $q_{j}^{\beta }$ that do not produce unexpectedly large DLT probabilities, while ensuring the production of predictive values similar to the DLT probabilities predicted under a prior distribution of γ with a larger SD (e.g., SD = 1500). This approach is somewhat complicated, but we confirmed that it achieves similar design performance to that obtained with a prior distribution of γ with a larger SD (see Web Appendix H). Similarly, GUMH‐E and Joe‐E with a prior distribution of γ presuming a large drug–drug interaction exhibited better performance (see Web Tables 11 and 12 in Web Appendix F).

Numerous dose‐finding designs for drug combinations have been proposed over the years, however, none of them are decidedly better than the other in terms of crucial outcome measures (e.g., the percentage of correct MTDC selection). Considering the growing popularity of combining anticancer drugs with different action mechanisms in cancer treatment, more complicated dose‐finding designs may be required in the future. In the absence of exceptional dose‐finding designs, development of dose‐finding designs with additional features, such as BOIN and Keyboard design (Pan *et al.*, 2020), which are easily implementable and exhibit comparable performance to the other designs, may be beneficial. In particular, as with copula‐type models, the proposed method exhibits distinctive additional advantages as dose‐finding designs as they are applicable to situations in which certain toxicities can be attributed to one of the two agents and they can also naturally handle binary DLT responses when either of the two drugs is administered as a single agent. Future studies are warranted to apply the proposed method to such situations. However, considering future expandability, the results obtained in this study could motivate the real‐world application of the proposed method in cases requiring the utilization of the properties of copula‐type models.

### OPEN RESEARCH BADGES

This article has earned an Open Materials badge for making publicly available the components of the research methodology needed to reproduce the reported procedure and analysis.

## Supporting information

Web Appendices and Tables referenced in Sections 2.2, 4.1, 4.2 and 5, as well as the simulation code are available with this paper at the Biometrics website on Wiley Online Library.

## Data Availability

Data sharing is not applicable to this article as no new data were created or analyzed in this paper.

## References

[biom13510-bib-0001] Ali, M. M. , Mikhail, N. N. and Haq, M. S. (1978) A class of bivariate distributions including the bivariate logistic. Journal of Multivariate Analysis, 8, 405–412.

[biom13510-bib-0002] Bedard, P. L. , Tabernero, J. , Janku, F. , Wainberg, Z. A. , Paz‐Ares, L. , Vansteenkiste, J. et al. (2015) A phase Ib dose‐escalation study of the oral Pan‐PI3K inhibitor Buparlisib (BKM120) in combination with the oral MEK1/2 inhibitor Trametinib (GSK1120212) in patients with selected advanced solid tumors. Clinical Cancer Research, 21, 730–738. 25500057 10.1158/1078-0432.CCR-14-1814

[biom13510-bib-0003] Cheung, Y. (2011) Dose Finding by the Continual Reassessment Method, Boca Raton, FL: CRC Press/Taylor & Francis Group.

[biom13510-bib-0004] Gandhi, L. , Bahleda, R. , Tolaney, S. M. , Kwak, E. L. , Cleary, J. M. , Pandya, S. S. et al. (2014) Phase I study of Neratinib in combination with Temsirolimus in patients with human epidermal growth factor receptor 2–dependent and other solid tumors. Journal of Clinical Oncology, 32, 68–75. 24323026 10.1200/JCO.2012.47.2787

[biom13510-bib-0005] Gasparini, M. (2013) General classes of multiple binary regression models in dose finding problems for combination therapies. Journal of the Royal Statistical Society: Series C (Applied Statistics), 62, 115–133.

[biom13510-bib-0006] Gasparini, M. , Bailey, S. and Neuenschwander, B. (2010) Correspondence: Bayesian dose finding in oncology for drug combinations by copula regression. Journal of the Royal Statistical Society: Series C (Applied Statistics), 59, 543–546.

[biom13510-bib-0007] Hirakawa, A. , Wages, N. A. , Sato, H. and Matsui, S. (2015) A comparative study of adaptive dose‐finding designs for phase I oncology trials of combination therapies. Statistics in Medicine, 34, 3194–3213. 25974405 10.1002/sim.6533 PMC4806394

[biom13510-bib-0008] Jimenez, J. L. , Tighiouart, M. and Gasparini, M. (2019) Cancer phase I trial design using drug combinations when a fraction of dose limiting toxicities is attributable to one or more agents. Biometrical Journal, 61, 319–332. 29808507 10.1002/bimj.201700166 PMC6261712

[biom13510-bib-0009] Lam, C. K. , Lin, R. and Yin, G. (2019) Non‐parametric overdose control for dose finding in drug combination trials. Journal of the Royal Statistical Society: Series C (Applied Statistics), 68, 1111–1130.

[biom13510-bib-0010] Lin, R. and Yin, G. (2016) Bootstrap aggregating continual reassessment method for dose finding in drug‐combination trials. The Annals of Applied Statistics, 10, 2349–2376.

[biom13510-bib-0011] Lin, R. and Yin, G. (2017) Bayesian optimal interval design for dose finding in drug‐combination trials. Statistical Methods in Medical Research, 26, 2155–2167. 26178591 10.1177/0962280215594494

[biom13510-bib-0012] Mander, A. P. and Sweeting, M. J. (2015) A product of independent beta probabilities dose escalation design for dual‐agent phase I trials. Statistics in Medicine, 34, 1261–1276. 25630638 10.1002/sim.6434 PMC4409822

[biom13510-bib-0013] Mozgunov, P. , Paoletti, X. and Jaki, T. (2021) A benchmark for dose‐finding studies with unknown ordering. Biostatistics, kxaa054. 10.1093/biostatistics/kxaa054 PMC9291639 33409536

[biom13510-bib-0014] Pan, H. , Lin, R. , Zhou, Y. and Yuan, Y. (2020) Keyboard design for phase I drug‐combination trials. Contemporary Clinical Trials, 92, 105972. 32151751 10.1016/j.cct.2020.105972

[biom13510-bib-0015] Riviere, M.‐K. , Yuan, Y. , Dubois, F. and Zohar, S. (2014) A Bayesian dose‐finding design for drug combination clinical trials based on the logistic model. Pharmaceutical Statistics, 13, 247–257. 24828456 10.1002/pst.1621

[biom13510-bib-0016] Simmet, V. , Eberst, L. , Marabelle, A. and Cassier, P. A. (2019) Immune checkpoint inhibitor‐based combinations: is dose escalation mandatory for phase I trials? Annals of Oncology, 30, 1751–1759. 31435659 10.1093/annonc/mdz286

[biom13510-bib-0017] Thall, P. F. , Millikan, R. E. , Mueller, P. and Lee, S.‐J. (2003) Dose‐finding with two agents in phase I oncology trials. Biometrics, 59, 487–496. 14601749 10.1111/1541-0420.00058

[biom13510-bib-0018] Wages, N. A. and Conaway, M. R. (2013) Specifications of a continual reassessment method design for phase I trials of combined drugs. Pharmaceutical Statistics, 12, 217–224. 23729323 10.1002/pst.1575 PMC3771354

[biom13510-bib-0019] Wages, N. A. , Conaway, M. R. and O'Quigley, J. (2011) Continual reassessment method for partial ordering. Biometrics, 67, 1555–1563. 21361888 10.1111/j.1541-0420.2011.01560.x PMC3141101

[biom13510-bib-0020] Wang, K. and Ivanova, A. (2005) Two‐dimensional dose finding in discrete dose space. Biometrics, 61, 217–222. 15737096 10.1111/j.0006-341X.2005.030540.x

[biom13510-bib-0021] Wheeler, G. M. , Sweeting, M. J. , Mander, A. P. , Lee, S. M. and Cheung, Y. K. K. (2017) Modelling semi‐attributable toxicity in dual‐agent phase I trials with non‐concurrent drug administration. Statistics in Medicine, 36, 225–241. 26891942 10.1002/sim.6912 PMC5157785

[biom13510-bib-0022] Yin, G. and Lin, R. (2015) Comments on “Competing designs for drug combination in phase I dose‐finding clinical trials” by M‐K. Riviere, F. Dubois, and S. Zohar. Statistics in Medicine, 34, 13–17. 25492615 10.1002/sim.6338

[biom13510-bib-0023] Yin, G. and Yuan, Y. (2009a) Bayesian dose finding in oncology for drug combinations by copula regression. Journal of the Royal Statistical Society: Series C (Applied Statistics), 58, 211–224.

[biom13510-bib-0024] Yin, G. and Yuan, Y. (2009b) A latent contingency table approach to dose finding for combinations of two agents. Biometrics, 65, 866–875. 18759848 10.1111/j.1541-0420.2008.01119.x

[biom13510-bib-0025] Yin, G. and Yuan, Y. (2010) Authors' response. Journal of the Royal Statistical Society: Series C (Applied Statistics), 59, 544–546.

