# Supplementary material for: Flexible use of copula‐type model for dose‐finding in drug combination clinical trials
Source: Biometrics. 2021 Aug 1;78(4):1651–61. doi: 10.1111/biom.13510 (PMC10393268; doi:10.1111/biom.13510)
Supplement: Supplementary file 1 — Web Appendices and Tables referenced in Sections 2.2, 4.1, 4.2 and 5, as well as the simulation code are available with this paper at the Biometrics website on Wiley Online Library. [file BIOM-78-1651-s001.zip › biom13510-sup-0001-SuppMat.pdf]

Supporting Information for "Flexible Use of Copula-type Model for Dose-finding in Drug Combination Clinical Trials," by

Koichi Hashizume[1,2,*], Jun Tshuchida[3,], Takashi Sozu[4,***]**

[1] Department of Information and Computer Technology, Graduate School of Engineering, Tokyo University of Science, Tokyo, Japan

[2] Global Biometrics and Data Science, Bristol Myers Squibb K.K., Tokyo, Japan

[3] Department of Culture and Information Science, Faculty of Culture and Information Science, Doshisha University, Kyoto, Japan

[4] Department of Information and Computer Technology, Faculty of Engineering, Tokyo University of Science, Tokyo, Japan

**email:* koichi.hashizume@bms.com

***email:* jtsuchid@mail.doshisha.ac.jp

****email:* sozu@rs.tus.ac.jp

This supplementary note includes the following: further characterization of the proposed method (Web Appendix A), additional results related to the simulations described in Sections 4.1 and 4.2 of the manuscript (Web Appendix B), additional numerical studies proving the clinical applicability of the proposed method is comparable to those of other competitive designs (Web Appendicies C, D, and E), additional results in cases of changing prior settings (Web Appendix F), the assessment of the bias of an estimator of toxicity probabilities for the original copula-type model vs. the proposed method (Web Appendix G), and the estimation method to find more appropriate estimate of γ (Web Appendix H).

Web Appendix A: Properties of a copula and the proposed method

In this section, we summarize the definitions and properties of a copula. Subsequently, proper-quasi copulas and the proposed method are characterized based on these definitions. Finally, the differences between a copula and the proposed method are explored and the influence of these differences on the estimation of toxicity probability in drug combination clinical trials is evaluated.

Definition: $\boldsymbol{I}^2$ denotes the unit square, i.e., the product $\boldsymbol{I} \times \boldsymbol{I}$, where $\boldsymbol{I}$ = [0,1]. A (two-dimensional) copula is a function C: $\boldsymbol{I}^2 \to \boldsymbol{I}$, such that

(A1) $C(0, a) = C(a, 0) = 0$ and $C(1, a) = C(a, 1) = a$ for all $a \in \boldsymbol{I}$.

(A2) C is 2-increasing: for $a, b, c, d \in \boldsymbol{I}$ with $a \leqslant b$ and $c \leqslant d$, V_C $([a, b] \times [c, d]) = C(b, d) - C(a, d) - C(b, c) + C(a, c) \geqslant 0$. The function, V_C, is referred to as the C-volume of the rectangle $[a, b] \times [c, d]$. If C satisfies A2, it also satisfies A3 and A4:

(A3) C is non-decreasing in each variable (i.e., for all u and v in $\boldsymbol{I}$, $\partial C(u, v)/\partial u$ and $\partial C(u, v)/\partial v$ are both non-negative)

(A4) C satisfies the following Lipschitz condition: for every a, b, c, d in $\boldsymbol{I}$ with $a \leqslant b$ and $c \leqslant d$, $|C(b, d) - C(a, c)| \leqslant |b - a| + |d - c|$.

The Fréchet–Hoeffding (F-H) bound is a combined consequence of A1, A3, and A4. Even though every copula is a quasi-copula, every quasi-copula is not a copula. Quasi-copulas that are not copulas are called proper quasi-copulas (Genest et al., 1999; Nelsen, 2006). Web Table 1 records the fulfillment of each condition of a copula by a proper quasi-copula and the proposed method.

Web Table 1: Relationships among copula, proper quasi-copula and the proposed method

Definitions and others	Copula	Proper Quasi-Copula	Proposal
A1 (admissibility cond.)	✓	✓	✓
A2 (2-increasing)	✓		
A3 (non-decreasing)	✓	✓	✓
A4 (Lipschitz)	✓	✓	
The restriction of F-H bound exists	✓	✓	No. By extending the parameter space of γ, F-H bound is extended. [a]
Proper distribution function	✓	✓	The regression model of π_{ij} in the proposed method is not a joint distribution function derived using a copula but merely one to estimate π_{ij}. Thus, it does not matter if the function is proper or not as a distribution function.

[a]Our proposal is to extend the parameter space of γ within the ranges satisfying the relaxed restriction that π_{ij} is within 0 to 1. γ is a dependent parameter in a copula-type model.

The proofs that the proposed method satisfies the conditions, A1 and A3, follow.

Proof. (A1) From the equation $C(u, v) = uv/(1 - \gamma(1 - u)(1 - v))$ of the AMH copula, it is readily apparent that γ, which appears in the denominator of $C(u, v)$, does not contribute to $C(u, v)$ when $u = 0$, $u = 1$, $v = 0$, and $v = 1$. Therefore, the AMH copula satisfies A1 irrespective of the parameter space of γ.

Proof. (A3) $\partial C(u, v)/\partial v$ of the AMH copula is given by $u(1-\gamma(1-u))/(1-\gamma(1-u)(1-v))^2$. This is non-negative in the proposed range, $[-\infty, 1]$. $\partial C(u, v)/\partial u$ is also non-negative. Therefore, the proposed method satisfies A3.

For the other copulas considered in the manuscript like Clayton and FGM, the extended parameter space of γ is dependent on u and v of $C(u, v)$. Thus, their fulfillment of A1 and A3 are not proved. A2 and A4 are not satisfied in ranges that deviate the original parameter space of γ. For example, when a = 0.65, b = 0.95, c = 0.95, and d = 0.97 under the $C(u, v)$ of the AMH copula with $\gamma = -100$, A2 is not satisfied (the value of the left-hand side of the inequality is -0.003). Further, for instance, when a = 0.85, b = 0.89, c = 0.78, and d = 0.83 under the $C(u, v)$ of the AMH copula with $\gamma = -100$, A4 is not satisfied (the value of $|C(b, d) - C(a, c)| - |b - a| + |d - c|$ is 0.013). Based on a similar way, it can be proved that GUMH and Joe copulas satisfy A1 and A3 and do not satisfy A2 and A4.

It is apparent from Web Table 1 that the proposed method is neither a copula nor a proper quasi-copula except for when γ is within its original parameter space. However, it still satisfies the conditions, A1 and A3. As described in the manuscript, fulfillment of A1 in modeling denotes that the joint toxicity probability, π_{ij}, is equal to the toxicity probability of one drug if the toxicity probability of the other drug within the combination is known to be zero. Furthermore, fulfillment of A3 implies that the toxicity of dual agents is monotonic and increases with the dose. Therefore, A1 and A3 are important conditions. On the contrary, A2 and A4 do not play important roles in the estimation of toxicity probability in drug combination clinical trials. Rather, by deviating from A2 and A4, the proposed method acquires broader F-H bounds than those of a copula, which enables flexible estimation of joint toxicity probability in drug combination trials. Overall, the discussion presented in this section clarifies the properties of the proposed method.

References

Genest, C., Molina, J. Q., Lallena, J. R., and Sempi, C. (1999). A characterization of quasicopulas. *Journal of Multivariate Analysis* **69,** 193–205.

Nelsen, R. B. (2006). *An Introduction to Copulas (Springer Series in Statistics).* Springer-Verlag, Berlin, Heidelberg.

Web Appendix B: Results of the outcome metrics for the safety aspects

This section presents the three remaining safety metrics related to the simulations described in Sections 4.1 and 4.2. Web Table 2 shows that the proposed method (COPULA-E: AMH-E, GUMH-E, and Joe-E) exhibited improvements over the original method (COPULA: AMH-O, GUMH-O, and Joe-O) with respect to each of the three safety metrics.

Web Table 2: Comparison between COPULA and COPULA-E in terms of the three outcome metrics for the safety aspects evaluated in Section 4.1. Scenarios highlighted in gray involve extreme synergistic toxicity. Avg represents the average across the 18 scenarios.

	Scenarios																		
Designs	1	2	3	4	5	6	7	8	9	10	11	12	13	14	15	16	17	18	Avg
	Overall percentage of observed toxicity																		
AMH-O	24	28	27	24	22	24	21	25	20	24	21	26	22	24	22	20	23	23	23
AMH-E	22	26	25	21	19	21	18	21	18	23	20	25	21	22	20	18	21	20	21
GUMH-O	24	29	28	24	23	24	22	25	20	25	21	27	23	25	22	20	24	23	24
GUMH-E	23	27	26	22	21	22	19	23	19	23	20	25	22	24	21	19	22	22	22
Joe-O	24	29	28	24	22	24	22	25	20	25	21	27	23	25	22	20	24	23	24
Joe-E	23	27	25	22	21	22	20	24	19	23	20	25	21	24	21	19	22	22	22
	Percentage of patients allocated to MTDC																		
AMH-O	29	54	26	13	27	23	13	10	21	36	45	9	20	18	17	4	22	16	22
AMH-E	28	59	26	21	31	23	15	19	20	36	45	20	22	22	23	11	24	13	25
GUMH-O	28	54	26	13	25	22	13	10	22	36	47	11	20	17	17	4	21	17	22
GUMH-E	28	57	25	18	27	22	15	17	21	37	46	15	20	17	21	6	18	14	24
Joe-O	29	54	28	12	25	22	12	10	22	37	49	11	20	16	18	4	21	18	23
Joe-E	27	56	26	13	27	24	13	15	20	39	42	10	20	15	20	6	18	17	23
	Percentage of patients allocated to OC																		
AMH-O	15	20	35	37	19	26	25	35	15	23	-	28	15	32	16	27	24	27	23
AMH-E	10	15	32	25	11	19	14	24	7	19	-	20	10	24	9	17	17	20	16
GUMH-O	17	20	37	37	22	27	27	36	16	25	-	29	16	33	17	27	27	28	25
GUMH-E	13	17	32	30	16	24	19	27	10	21	-	22	11	28	13	22	22	23	19
Joe-O	16	21	35	38	21	26	26	36	16	24	-	28	15	33	17	27	25	26	24
Joe-E	13	16	31	32	17	23	21	29	10	19	-	24	11	30	15	23	23	24	20

Web Table 3 shows that the percentage of patients allocated to true MTDC was observed to be similar for all the dose-finding designs excluding TDM. In addition, AMH-E and LOGI exhibited lower percentages of patients allocated to OC and overall percentages of observed toxicity compared to TDM and BOIN in all 18 scenarios and compared to POCRM in most

scenarios, suggesting that AMH-E and LOGI make decisions regarding dose escalation less frequently compared to TDM, POCRM, and BOIN.

Web Table 3: Comparison between AMH-E and existing competitive dose-finding designs in terms of the three outcome metrics for the safety aspects evaluated in Section 4.2. Scenarios highlighted in gray involve extreme synergistic toxicity. Avg represents the average across the 18 scenarios.

	Scenarios																		
Designs	1	2	3	4	5	6	7	8	9	10	11	12	13	14	15	16	17	18	Avg
	Overall percentage of observed toxicity																		
AMH-E	23	27	25	22	19	21	18	22	18	24	20	25	21	23	20	18	21	21	22
LOGI	23	29	27	20	19	22	18	21	18	24	20	26	21	22	19	17	22	20	22
TDM	24	29	28	24	22	24	21	26	20	25	21	27	23	25	22	21	24	23	24
POCRM	24	34	31	21	18	22	17	21	20	27	22	31	21	22	20	15	23	21	23
BOIN	26	30	29	24	23	24	22	25	22	26	23	29	23	25	23	22	24	23	25
	Percentage of patients allocated to MTDC																		
AMH-E	28	59	26	21	31	23	15	19	20	36	45	20	22	22	23	11	24	13	25
LOGI	33	63	29	21	32	27	14	20	19	38	44	18	18	24	25	11	21	14	26
TDM	29	52	25	13	25	22	13	10	21	34	48	11	20	17	18	4	21	17	22
POCRM	38	54	27	16	31	27	17	22	29	48	54	22	24	21	24	14	22	15	28
BOIN	32	52	24	20	38	29	21	20	26	32	56	15	23	27	22	13	19	14	27
	Percentage of patients allocated to OC																		
AMH-E	10	15	32	25	11	19	14	24	7	19	-	20	10	24	9	17	17	20	16
LOGI	9	16	34	23	10	17	14	23	6	18	-	21	7	22	7	15	16	17	15
TDM	17	23	36	36	21	27	26	35	14	24	-	28	17	31	16	27	25	27	24
POCRM	12	32	45	27	9	19	10	22	8	23	-	39	6	24	9	8	22	20	19
BOIN	19	25	41	32	16	24	19	29	14	30	-	33	13	26	27	25	28	24	24

Web Appendix C: Comparison of each dose-finding design with the nonparametric optimal benchmark

In this section, we evaluate the absolute performance of dose-finding designs featured in the manuscript using the nonparametric optimal benchmark proposed by Mozgunov et al. (2021). Web Table 4 records the MTDC selections (%) of the benchmark and the ratio of the MTDC selection (%) of each design to that of the benchmark.

Whereas the proposed method, AMH-E, exhibits a ratio of at least 83% in 17 out of the 18 scenarios, the existing method, AMH-O, exhibits a ratio above 81% in only 7 out of the

18 scenarios. Notably, although AMH-E and AMH-O utilize identical simulation settings except for the parameter space of γ (e.g., skeletons, escalation rules, etc.), AMH-E exhibits a more stable performance with respect to the benchmark than AMH-O. As with AMH-E against AMH-O, the proposed methods, GUMH-E and Joe-E, exhibit better performance than GUMH-O and Joe-O, respectively. The number of scenarios with ratios less than 80% for LOGI, TDM, POCRM, and BOIN are 2, 12, 5, and 3, respectively. The performance of the designs except for TDM and AMH-O are also observed to be comparable in the evaluation based on the benchmark.

Web Table 4: Comparison of each dose-finding design with the nonparametric optimal benchmark. Scenarios highlighted in gray denote the existence of extreme synergistic toxicity. Avg represents the average over the 18 scenarios. AMH-E, GUMH-E, and Joe-E are the proposed method (COPULA-E) and AMH-O, GUMH-O, and Joe-O are the original method (COPULA).

	Scenarios																		
Designs	1	2	3	4	5	6	7	8	9	10	11	12	13	14	15	16	17	18	Avg
	MTDC selections (%)																		
Benchmark	49	83	49	37	76	42	36	21	47	46	90	43	38	41	35	21	39	26	46
	Ratio of MTDC selections (%) with respect to benchmark																		
AMH-O	86	89	78	65	57	67	75	81	81	96	88	51	92	76	60	24	62	69	74
AMH-E	98	101	69	105	87	114	92	157	83	115	86	91	95	100	109	105	95	85	96
GUMH-O	80	84	78	70	42	57	72	86	83	78	93	56	82	66	43	24	54	58	70
GUMH-E	90	99	90	105	76	105	86	152	87	111	91	79	92	76	97	62	72	85	89
Joe-O	84	83	82	68	47	57	72	76	79	85	97	58	87	71	46	19	56	73	72
Joe-E	88	94	94	70	66	93	83	124	87	120	86	53	92	63	77	52	64	88	83
LOGI	106	102	84	95	95	112	103	181	87	104	88	95	89	105	120	119	77	69	98
TDM	94	87	78	49	54	69	64	62	81	107	91	74	79	56	74	14	77	104	74
POCRM	118	83	65	54	91	107	97	157	102	135	87	77	113	90	103	129	79	77	93
BOIN	94	98	71	81	96	114	117	143	94	113	88	70	100	102	100	110	69	85	93

References

Mozgunov, P., Paoletti, X., and Jaki, T. (2021). A benchmark for dose-finding studies with unknown ordering. *Biostatistics.*

Web Appendix D: Assessment of dose-finding designs under the application of the stopping rule

The design using a copula-type model proposed by Yin and Yuan (2009a,b) included the stopping rule strategy and demonstrated its satisfactory applicability in scenarios where all dose combinations are considered to be overly toxic. In this section, we evaluate the operating characteristics of designs based on the original copula-type models (COPULA: AMH-O, GUMH-O, and Joe-O) and the proposed methods (COPULA-E: AMH-E, GUMH-E, and Joe-E) under the application of the stopping rule. As described in the manuscript, the trial can be terminated at an early stage for safety if even the lowest dose combination is overly toxic, as indicated by $\Pr(\pi_{11} > \phi \mid \mathrm{D}) > \lambda$. ϕ and D denote the target toxicity rate and the cumulative data, respectively. We set $\lambda = 0.9$ for all the designs. We conduct simulation studies under the four overly toxic scenarios listed in Web Table 5 besides the 18 scenarios listed in Table 3 of the manuscript. Unless otherwise noted, we use identical simulation settings (e.g., the number of simulations, the total sample size, etc.) to those used in the manuscript.

Web Table 5: Scenarios where all dose combinations are overly toxic

		A									
		1	2	3	4	5	1	2	3	4	5
		Scenario 21					Scenario 22				
	4						0.70	0.80	0.90	0.95	
	3	0.80	0.90	0.95			0.60	0.70	0.80	0.90	
	2	0.70	0.80	0.90			0.55	0.60	0.70	0.80	
	1	0.60	0.70	0.80			0.50	0.55	0.60	0.70	
B											
		Scenario 23					Scenario 24				
	3						0.65	0.75	0.85	0.95	0.99
	2	0.60	0.70	0.80	0.90		0.60	0.70	0.80	0.90	0.95
	1	0.50	0.60	0.70	0.80		0.55	0.60	0.70	0.80	0.85

Web Table 6 verifies that the proposed method is equipped with the capability to terminate a trial satisfactorily in overly toxic scenarios.

Web Table 6: Evaluation of the stopping rule in scenarios where all dose combinations are overly toxic.

	Scenarios				
Designs	21	22	23	24	Avg
	Trial termination (%)				
AMH-E	99	89	88	95	92
AMH-O	99	84	88	94	91
GUMH-E	98	90	90	94	93
GUMH-O	98	85	88	93	91
Joe-E	99	91	94	98	95
Joe-O	98	85	87	93	90
	Average number of treated patients				
AMH-E	6	10	10	8	9
AMH-O	8	14	13	11	11
GUMH-E	8	12	11	11	10
GUMH-O	8	14	13	11	11
Joe-E	6	10	9	7	8
Joe-O	9	14	13	11	12

Further, the proposed method is observed to be capable of terminating the trials based on an average number of treated patients that is lower than that of the original design. Trial termination (%) denotes the percentage of trials that were successfully stopped.

In addition, Web Table 7 reveals that, even in normal scenarios where at least one MTDC exists within each dose combination, the proposed method performs well irrespective of whether the stopping rule is implemented. The dose combinations with toxicity probabilities greater than or equal to 0.35 were defined to be overdose combinations (OCs). Larger values of the accuracy index correspond to higher dose-finding accuracies.

References

Yin, G. and Yuan, Y. (2009a). Bayesian dose finding in oncology for drug combinations by copula regression. *Journal of the Royal Statistical Society: Series C (Applied Statistics)* **58,** 211–224.

Yin, G. and Yuan, Y. (2009b). A latent contingency table approach to dose finding for combinations of two agents. *Biometrics* **65,** 866–875.

Web Table 7: Values of the four outcome metrics for COPULA vs. COPULA-E under the application of the stopping rule. Scenarios highlighted in gray represent scenarios involving extreme synergistic toxicity. Avg represents the average across the 18 scenarios.

	Scenarios																		
Designs	1	2	3	4	5	6	7	8	9	10	11	12	13	14	15	16	17	18	Avg
	MTDC selections (%)																		
AMH-E	46	82	31	39	68	47	33	31	38	53	72	37	35	43	36	20	37	21	43
AMH-O	46	73	39	24	43	28	25	18	42	41	81	26	34	29	18	7	24	17	34
GUMH-E	46	79	43	34	61	44	31	33	42	49	77	32	36	30	32	15	27	23	41
GUMH-O	39	70	40	25	35	29	26	19	39	38	83	25	32	26	17	6	20	17	33
Joe-E	46	76	44	27	50	40	31	26	38	53	74	23	38	27	26	12	27	23	38
Joe-O	38	67	42	27	34	24	27	17	39	38	83	23	30	26	18	6	20	18	32
	OC selections (%)																		
AMH-E	16	14	47	40	21	31	33	41	14	24	-	19	19	32	20	40	29	35	28
AMH-O	25	15	44	66	42	50	54	55	32	41	-	29	34	56	41	67	46	56	44
GUMH-E	20	12	39	50	28	34	42	41	23	30	-	22	22	42	24	49	40	41	33
GUMH-O	33	15	40	67	48	53	55	55	38	45	-	29	38	56	44	69	50	58	47
Joe-E	20	13	41	58	39	44	49	54	25	31	-	30	26	52	35	61	41	46	39
Joe-O	31	15	38	66	50	55	54	58	39	46	-	31	37	58	45	68	51	57	47
	Accuracy index																		
AMH-E	38	85	58	56	65	57	56	58	43	63	30	68	57	58	56	45	41	41	54
AMH-O	30	72	49	33	32	31	35	36	47	45	37	52	42	38	34	24	27	28	38
GUMH-E	36	81	60	50	57	52	53	57	47	59	34	61	56	49	52	40	34	39	51
GUMH-O	19	69	48	28	18	28	27	36	45	41	39	52	38	32	27	15	21	24	34
Joe-E	35	76	57	42	43	49	43	47	43	59	34	53	54	42	43	32	33	36	46
Joe-O	21	66	50	31	20	27	29	35	47	40	41	49	37	30	28	17	20	28	34
	Trial termination (%)																		
AMH-E	3	1	1	0	0	0	0	0	1	0	3	3	1	0	0	0	1	0	1
AMH-O	0	0	0	0	0	0	0	0	0	0	1	1	0	0	0	0	0	0	0
GUMH-E	2	1	1	0	0	0	0	0	1	0	1	2	0	0	0	0	1	0	0
GUMH-O	0	0	0	0	0	0	0	0	0	0	0	0	0	0	0	0	0	0	0
Joe-E	3	1	1	0	0	1	0	0	1	0	4	2	1	0	0	0	0	0	1
Joe-O	0	0	0	0	0	0	0	0	0	0	0	0	0	0	0	0	0	0	0

Web Appendix E: Operating characteristics of the proposed method in scenarios where all dose combinations exhibit extremely low toxicity

In order to assess the behavior of the proposed method in scenarios where all dose combinations exhibit extremely low toxicity, numerical studies are conducted in this section in the four scenarios listed in Web Table 8.

Web Table 8: Scenarios where all dose combinations exhibit extremely low toxicity

		A									
		1	2	3	4	5	1	2	3	4	5
		Scenario 31					Scenario 32				
	3	0.05	0.07	0.10			0.04	0.10	0.20		
	2	0.03	0.05	0.07			0.02	0.04	0.10		
	1	0.01	0.03	0.05			0.01	0.02	0.04		
B											
		Scenario 33					Scenario 34				
	2	0.03	0.05	0.07	0.10		0.02	0.05	0.10	0.20	
	1	0.01	0.03	0.05	0.07		0.01	0.02	0.05	0.10	

We evaluate the designs based on the original copula-type models (COPULA: AMH-O, GUMH-O, and Joe-O) and the proposed methods (COPULA-E: AMH-E, GUMH-E, and Joe-E). The outcome metric, "the average number of patients allocated to the maximum dose combinations", is used to assess the capability of the proposed method to escalate the dose smoothly. Unless otherwise noted, we use the same simulation settings (e.g., the number of simulations, the total sample size, etc.) as those recorded in the manuscript. Web Table 9 reveals that the performances of the original and the proposed methods are similar in each scenario and each model, suggesting that the proposed method has the capability of escalating the dose as smoothly as the designs based on the original copula-type models.

Web Table 9: Operating characteristics of the proposed and the original methods in scenarios where all dose combinations exhibit extremely low toxicity.

	Scenarios				
Designs	31	32	33	34	Avg
	Average number of patients allocated to maximum dose combinations				
AMH-E	8.2	7.4	9.4	8.8	8.5
AMH-O	9.0	8.9	10.6	10.3	9.7
GUMH-E	8.7	8.3	10.0	9.5	9.1
GUMH-O	9.4	8.7	10.7	10.4	9.8
Joe-E	9.0	8.1	9.9	9.5	9.1
Joe-O	9.1	8.8	10.7	10.7	9.8

Web Appendix F: Results of varying the prior settings of γ for COPULA-E

This section provides additional results in cases of changing prior settings for the proposed method (COPULA-E: AMH-E, GUMH-E, and Joe-E). The simulation results suggest that any model with a prior distribution of γ presuming a large drug-drug interaction exhibited better performance.

Web Table 10: Values of the three primary outcome metrics for AMH-based COPULA-E (AMH-E) under different prior settings of γ (the standard deviation is varied). Scenarios highlighted in gray involve extreme synergistic toxicity. Avg represents the average across the 18 scenarios.

AMH-E	Scenarios																	
(SD)	1	2	3	4	5	6	7	8	9	10	11	12	13	14	15	16	17	18	Avg
	MTDC selections (%)																		
50	43	82	40	27	61	45	27	21	39	56	78	32	36	30	32	11	31	27	40
100	44	81	43	35	62	45	32	28	42	53	76	33	38	28	35	12	27	24	41
500	45	83	37	37	66	47	30	28	43	51	77	39	38	31	37	16	31	27	42
1000	48	81	36	36	66	47	30	31	41	49	78	40	35	36	40	17	36	27	43
1500	48	84	34	39	66	48	33	33	39	53	77	39	36	41	38	22	37	22	44
3000	44	83	35	40	70	46	31	24	42	55	76	43	39	44	40	20	39	20	44
	OC selections (%)																		
50	19	11	44	50	28	32	51	50	27	27	-	22	24	43	25	53	39	44	35
100	20	13	43	46	28	32	42	43	23	27	-	23	23	37	26	50	40	43	33
500	19	13	48	44	23	29	41	40	18	26	-	19	20	34	21	44	34	37	30
1000	16	16	46	42	21	31	35	41	16	27	-	20	19	36	20	43	32	35	29
1500	15	13	48	41	20	26	31	39	15	25	-	20	20	33	19	41	29	35	28
3000	14	14	45	41	19	29	36	42	12	24	-	19	18	33	18	37	27	33	27
	Accuracy Index (AI)																		
50	28	83	60	45	57	52	47	50	44	64	33	60	52	47	54	39	37	43	50
100	31	82	63	52	59	53	52	55	46	62	31	60	57	49	54	38	33	42	51
500	32	85	62	54	63	56	52	57	48	61	32	66	59	52	58	43	36	44	53
1000	36	83	62	54	63	56	53	58	45	59	34	66	57	54	58	44	38	44	54
1500	36	86	60	57	64	57	57	59	44	64	32	67	57	58	59	47	39	40	55
3000	32	85	60	57	68	56	56	55	47	65	32	70	61	59	59	47	40	40	55

Web Table 11: Values of the three primary outcome metrics for GUMH-based COPULA-E (GUMH-E) under different prior settings of γ (the parameters of shape and scale are varied). Scenarios highlighted in gray involve extreme synergistic toxicity. Avg represents the average across the 18 scenarios.

GUMH-E (Shape, Scale)	Scenarios																		
	1	2	3	4	5	6	7	8	9	10	11	12	13	14	15	16	17	18	Avg
	MTDC selections (%)																		
(0.75,0.75)	43	77	42	26	58	44	33	25	38	54	78	29	37	29	30	12	26	22	39
(0.5,0.5)	44	82	44	39	58	44	31	32	41	51	82	34	35	31	34	13	28	22	41
(1,0.2)	46	82	41	40	61	47	33	31	39	51	77	34	36	30	36	13	28	28	42
(0.5,0.2)	45	79	36	37	67	46	36	32	40	53	77	37	39	35	35	16	36	25	43
	OC selections (%)																		
(0.75,0.75)	21	12	39	56	29	37	47	46	30	29	-	25	25	48	30	55	42	46	36
(0.5,0.5)	20	12	38	47	31	39	43	44	22	31	-	23	22	40	25	49	39	44	34
(1,0.2)	17	12	43	45	27	32	38	42	17	26	-	21	19	39	23	46	40	38	31
(0.5,0.2)	16	15	47	43	22	29	33	39	18	25	-	21	18	38	21	44	32	36	29
	Accuracy Index (AI)																		
(0.75,0.75)	29	77	56	44	55	49	50	51	43	61	33	56	53	45	49	37	31	39	48
(0.5,0.5)	30	83	61	56	54	53	50	57	46	60	39	60	54	48	54	40	33	38	51
(1,0.2)	34	84	62	55	56	55	53	57	43	61	31	61	56	49	55	41	35	45	52
(0.5,0.2)	34	81	61	53	63	55	55	57	44	62	33	63	59	53	55	43	36	41	53

Web Table 12: Values of the three primary outcome metrics for Joe-based COPULA-E (Joe-E) under different prior settings of γ (the parameters of shape and scale are varied). Scenarios highlighted in gray involve extreme synergistic toxicity. Avg represents the average across the 18 scenarios.

Joe-E (Shape, Scale)	Scenarios																		
	1	2	3	4	5	6	7	8	9	10	11	12	13	14	15	16	17	18	Avg
	MTDC selections (%)																		
(0.75,0.75)	42	77	46	20	43	39	31	23	35	52	78	26	32	29	27	8	28	19	36
(0.5,0.5)	43	78	46	26	50	39	30	26	41	55	77	23	35	26	27	11	25	23	38
(1,0.2)	46	76	45	30	52	39	29	33	40	50	76	23	35	28	27	12	28	24	38
(0.5,0.2)	43	78	43	35	52	44	31	39	38	53	75	24	35	24	29	12	23	25	39
	OC selections (%)																		
(0.75,0.75)	26	15	38	66	44	43	47	54	32	31	-	30	28	51	35	62	43	52	41
(0.5,0.5)	22	13	41	59	41	46	50	51	26	31	-	31	24	52	34	60	42	46	39
(1,0.2)	21	16	41	56	38	46	50	51	22	30	-	29	26	51	34	55	38	46	38
(0.5,0.2)	19	15	41	52	37	41	46	47	21	30	-	31	23	51	29	57	38	44	37
	Accuracy Index (AI)																		
(0.75,0.75)	25	77	58	37	35	45	43	45	40	58	32	52	48	41	44	29	30	34	43
(0.5,0.5)	31	78	58	41	42	47	44	47	45	62	30	51	51	41	46	29	30	37	45
(1,0.2)	32	76	59	47	44	49	41	52	44	58	31	51	51	43	45	34	32	37	46
(0.5,0.2)	28	78	57	52	46	52	43	56	43	60	29	51	53	42	51	34	28	38	47

Web Appendix G: Evaluation of the bias of an estimator of toxicity probabilities

In this section, we evaluate the bias of an estimator of toxicity probabilities using the data obtained from the numerical studies in the 18 scenarios presented in the manuscript. We use $\hat{\pi}_{ij} - \pi_{ij}$ for the evaluation of the bias. $\hat{\pi}_{ij}$ denotes the predicted toxicity probability obtained after the exhaustion of the maximum sample size in a simulated trial. For all dose combinations including unassigned dose combinations, $\hat{\pi}_{ij} - \pi_{ij}$ is calculated for each scenario, trial, and dose combination (e.g., in the case of nine dose combinations to be assessed and 1000 simulations, 9000 records are obtained). Scenarios are grouped into synergistic toxicity scenarios (Scenarios 4, 5, 6, 7, 8, 14, 15, and 16), non-synergistic toxicity scenarios, and all 18 scenarios. The calculated $\hat{\pi}_{ij} - \pi_{ij}$ is visualized using a box plot with the intervals, π_{ij}: $\pi_{ij} \leqslant 0.10$, $0.10 < \pi_{ij} \leqslant 0.25$, $0.25 < \pi_{ij} \leqslant 0.35$, $\pi_{ij} > 0.35$, and all. The biases of the original methods (AMH-O) and proposed method (AMH-E) are then compared.

Web Figure 1 illustrates that the original copula-type models tend to overestimate toxicity probabilities in dose combinations with lower toxicity probabilities (i.e., $\pi_{ij} < 0.10$) and underestimate them in dose combinations with higher toxicity probabilities (i.e., $\pi_{ij} > 0.35$), compared to the proposed method. This underestimation induced the higher overdose selection (%) observed in the numerical studies presented in the manuscript. Further, the biases of the original copula-type models corresponding to all dose combinations were larger on average than that of the proposed method irrespective of the presence of extreme synergistic toxicity. However, this was especially true in scenarios with extreme synergistic toxicity. The aforementioned observations suggest that the proposed method is capable of estimating toxicity probabilities with less bias on average than the original method.

For AMH-E, an analogous comparison is performed under different prior settings of γ: SD = 50, 100, 500, 1000, 1500 and 3000 in a truncated N(0, SD)$_{[-\infty,1]}$. The corresponding results

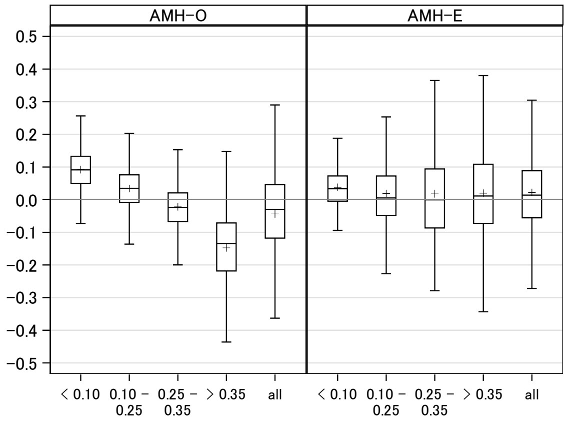

(a) Non-synergistic toxicity scenarios (10 scenarios)

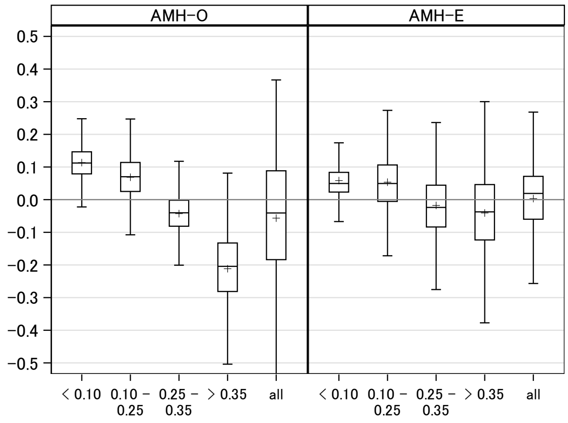

(b) Synergistic toxicity scenarios (8 scenarios)

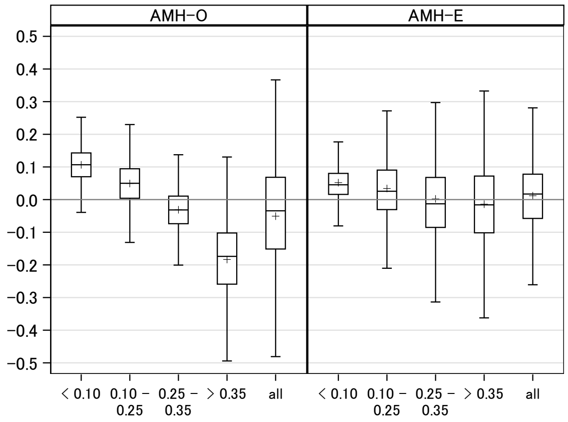

(c) All 18 scenarios

Web Figure 1: Comparison of the proposed method (AMH-E) and original method (AMH-O) in terms of the bias of an estimator of toxicity probability. Y-axis represents $\hat{\pi}_{ij} - \pi_{ij}$ (predicted value − true toxicity probability). X-axis represents classification of true toxicity probabilities.

are presented in Web Figure 2. It appears that bias decreases when the SD values increase. This is consistent with the performance presented in Web Table 10 in which the average MTDC selection percentage over 18 scenarios increases with increasing the SD values. Thus, somewhat larger SD values are preferable to specify a prior γ as described in the manuscript.

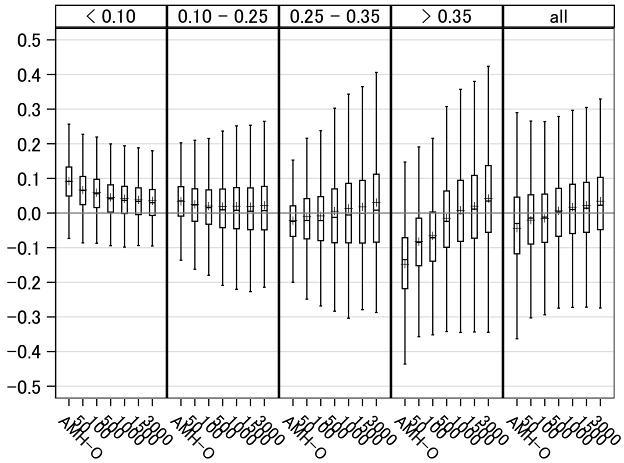

(a) Non-synergistic toxicity scenarios (10 scenarios)

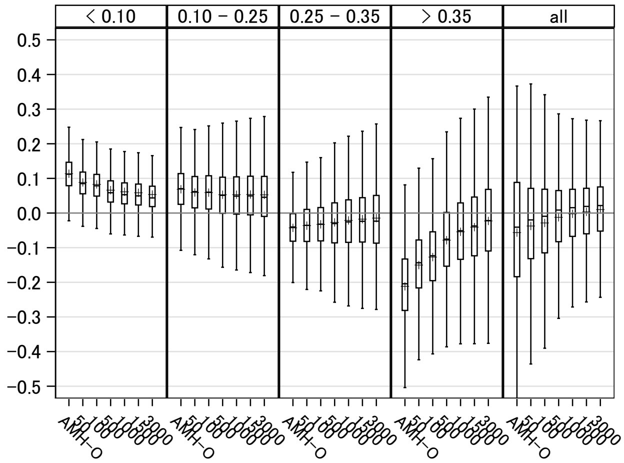

(b) Synergistic toxicity scenarios (8 scenarios)

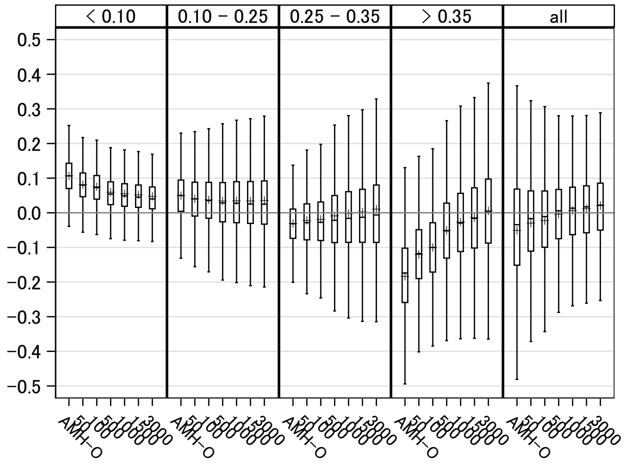

(c) All 18 scenarios

Web Figure 2: Comparison of the proposed methods applying the different SD to prior γ in terms of the bias of an estimator of the toxicity probability. Y-axis represents $\hat{\pi}_{ij} - \pi_{ij}$ (predicted value − true toxicity probability). X-axis represents specified SD for a prior distribution of γ. Header group represents classification of true toxicity probabilities.

Web Appendix H: The exploration of more appropriate estimates of γ in AMH-based COPULA-E (AMH-E)

The findings from Web Appendices F and G suggest that the bias of an estimator of DLT probability decreases and the MTDC selection percentage improves with an increase in the standard deviation (SD) of a prior distribution of γ in AMH-E. However, applying large negative values to γ produces unexpectedly large DLT probabilities from very small marginal probabilities. Note that γ takes larger negative values with an increase in the SD for a prior distribution of γ, as we assume a truncated normal distribution with an upper bound of 1.

In this section, we provide a method to seek more appropriate sets of γ, p_i^α and q_j^β that do not produce unexpectedly large DLT probabilities while ensuring the production of predictive values similar to the DLT probabilities predicted under a prior distribution of γ with a larger SD (e.g., SD = 1500). Web Table 13 provides an illustrative example showing two matrices with similar toxicity probabilities even though the values of γ differ significantly. Considering the value of marginal probabilities, the value of γ in Case 2 is more appropriate than that of Case 1 in practical situations.

Web Table 13: Similar toxicity probabilities produced by AMH-E with different γ values. DLT probabilities are calculated from the fixed value of γ, p_i^α, and q_j^β in the table.

		A					
		1	2	3	1	2	3
		Case 1 (γ = -1000)			Case 2 (γ = -25)		
	1	0.02	0.06	0.14	0.00	0.06	0.15
B	2	0.07	0.17	0.34	0.06	0.17	0.34
	3	0.15	0.34	0.56	0.16	0.34	0.54
		$p_1^\alpha, p_2^\alpha, p_3^\alpha$ = (0.00, 0.01, 0.02)			$p_1^\alpha, p_2^\alpha, p_3^\alpha$ = (0.00, 0.05, 0.14)		
		$q_1^\beta, q_2^\beta, q_3^\beta$ = (0.01, 0.02, 0.05)			$q_1^\beta, q_2^\beta, q_3^\beta$ = (0.00, 0.06, 0.16)		

The following is the proposed procedure for estimating γ like Case 2 above. Each time the data is updated, this procedure is executed.

(1) Estimate π_{ij} in AMH-E with γ assuming a prior distribution of truncated N(0,σ^2)$_{[-\infty,1]}$.

Set σ to a large value (e.g., 1500). Use the same conditions as the manuscript for the other settings (e.g., prior setting of α and β).

(2) Let the predictive values obtained from (1) be $\hat{\pi}_{ij}$.

(3) Provide a weight for each $\hat{\pi}_{ij}$ for all i and j. For example, if three subjects are treated at the dose combination (s, t), provide a weight of 3 for $\hat{\pi}_{st}$. Provide a weight of 1 for an untreated dose combination.

(4) Explore the sets of γ, p_i, and q_j that can produce similar predictive results to (1), following the steps below.

Step 1: Set γ to the fixed value of 1 (i.e., start from the upper bound of γ).

Step 2: Estimate the parameters of x_i and y_j of the following AMH-based model with non-linear optimization. The objective variable of $\hat{\pi}_{ij}$ weighted in (3) is considered as a continuous variable, and γ is treated as a fixed value. Let the obtained predictive values of $\hat{\pi}_{ij}$ be $\hat{\pi}^*_{ij}$.

$$\hat{\pi}_{ij} = 1 - (1 - x_i)(1 - y_i)/(1 - \gamma x_i y_i)$$

Step 3: Calculate Root Mean Square Error (RMSE).

$$\text{RMSE} = \sqrt{\sum_{i=1}^{I}\sum_{j=1}^{J}(\hat{\pi}_{ij} - \hat{\pi}^*_{ij})^2/(I \times J)},$$

where I and J are the maximum dose levels of drug A and drug B, respectively.

Step 4: If RMSE < 0.01, proceed with Step 5. Otherwise, decrease the current value of γ by 1 and return to Step 2.

Step 5: Refer to x_i, y_j, and γ at this point as x^*_i, y^*_j, and γ^* that can produce the similar predictive values to (1).

(5) Obtain standardized p^*_i by calculating $(x^*_i)^{(\log(p_k)/\log(x^*_k))}$, where k is determined by rounding $I/2$. p_k is the original skeleton value. With this calculation, when $I = 3$ and $p_2 = 0.20$,

p_2^* becomes 0.20. Obtain standardized q_j^* in the same manner. This standardization is performed to obtain a similar posterior distribution to those of (1).

(6) Replace p_i and q_j in the AMH-E with p_i^* and q_j^*, respectively, then estimate π_{ij} again using AMH-E with p_i^*, q_j^*, and γ assuming a prior distribution of N(γ^*, SD=$\gamma^*/3$, upper =1).

The aforementioned estimation procedure takes advantage of the fact that AMH-E enables flexible estimation with a decrease in the value of γ. This model using the estimation procedure is referred to as AMH-E2 hereafter. We compare the performances of AMH-E and AMH-E2 in the 18 scenarios listed in Table 3 of the manuscript. The SD values of 500, 1500, and 3000 are evaluated for a prior distribution of γ. The "SD" in AMH-E2 implies SD at the first estimation in the procedure. Web Tables 14 and 15 show the values of the three outcome metrics for the design efficiency and averaged posterior mean of γ. The simulation results reveal that AMH-E2 demonstrates a similar performance to AMH-E for the three outcomes. More importantly, the averaged posterior mean of γ is successfully reduced using AMH-E2. As described in the manuscript, if the estimates of the marginal probabilities are critical, using the proposed estimation procedure will be one of the options.

Web Table 14: Values of the three outcome metrics for AMH-E and AMH-E2 under different prior settings of γ. 500E and 500E2 represent AMH-E and AMH-E2 for a prior of γ with SD=500, respectively. Scenarios highlighted in gray denote the existence of extreme synergistic toxicity. Avg represents the average over the 18 scenarios.

	Scenarios																		
SD	1	2	3	4	5	6	7	8	9	10	11	12	13	14	15	16	17	18	Avg
	MTDC selections (%)																		
500E	45	83	37	37	66	47	30	28	43	51	77	39	38	31	37	16	31	27	42
500E2	48	85	41	36	65	47	29	29	43	61	78	42	32	38	41	15	36	27	44
1500E	48	84	34	39	66	48	33	33	39	53	77	39	36	41	38	22	37	22	44
1500E2	47	85	49	34	68	43	32	28	41	61	77	40	35	42	36	14	35	25	44
3000E	44	83	35	40	70	46	31	24	42	55	76	43	39	44	40	20	39	20	44
3000E2	48	87	45	40	67	49	37	36	40	60	76	39	32	41	40	15	30	28	45
	OC selections (%)																		
500E	19	13	48	44	23	29	41	40	18	26	-	19	20	34	21	44	34	37	30
500E2	16	12	41	42	23	30	41	41	17	18	-	24	20	35	20	47	29	36	29
1500E	15	13	48	41	20	26	31	39	15	25	-	20	20	33	19	41	29	35	28
1500E2	14	12	37	42	18	30	37	39	16	17	-	25	16	30	17	45	27	35	27
3000E	14	14	45	41	19	29	36	42	12	24	-	19	18	33	18	37	27	33	27
3000E2	14	12	39	37	18	28	32	35	15	16	-	25	17	31	15	41	31	31	26
	Accuracy index																		
500E	32	85	62	54	63	56	52	57	48	61	32	66	59	52	58	43	36	44	53
500E2	37	86	59	51	63	55	52	54	47	70	34	69	54	54	59	43	40	43	54
1500E	36	86	60	57	64	57	57	59	44	64	32	67	57	58	59	47	39	40	55
1500E2	36	87	64	51	66	52	56	55	45	71	33	69	58	57	58	44	39	43	55
3000E	32	85	60	57	68	56	56	55	47	65	32	70	61	59	59	47	40	40	55
3000E2	38	88	62	55	65	58	59	61	43	70	32	69	56	58	59	43	36	43	55

Web Table 15: Averaged posterior mean of γ for AMH-E and AMH-E2 under different prior settings of γ. 500E and 500E2 represent AMH-E and AMH-E2 for a prior of γ with SD=500, respectively. Scenarios highlighted in gray denote the existence of extreme synergistic toxicity. Avg represents the average of the 18 scenarios.

	Designs					
Scenarios	500E	500E2	1500E	1500E2	3000E	3000E2
	Averaged posterior mean of γ					
1	-354	-6	-1049	-9	-2070	-10
2	-379	-4	-1124	-6	-2210	-7
3	-373	-6	-1099	-8	-2191	-10
4	-376	-12	-1100	-18	-2167	-21
5	-383	-12	-1116	-17	-2199	-20
6	-371	-9	-1090	-13	-2152	-15
7	-377	-11	-1098	-16	-2183	-20
8	-388	-11	-1139	-15	-2229	-17
9	-345	-9	-1016	-13	-2017	-16
10	-369	-9	-1083	-12	-2131	-14
11	-347	-7	-1021	-10	-2041	-11
12	-370	-5	-1097	-6	-2167	-8
13	-360	-8	-1061	-12	-2098	-14
14	-390	-11	-1136	-15	-2234	-18
15	-368	-10	-1083	-15	-2156	-18
16	-370	-12	-1088	-17	-2146	-22
17	-359	-7	-1055	-11	-2090	-13
18	-360	-8	-1059	-12	-2104	-14
Avg	-369	-9	-1084	-13	-2144	-15
